# Should We Work Smarter or Harder for Our Health? A Comparison of Intensity and Domain-Based Time-Use Compositions and Their Associations With Cognitive and Cardiometabolic Health

**DOI:** 10.1093/gerona/glae233

**Published:** 2024-09-19

**Authors:** Maddison L Mellow, Dorothea Dumuid, Alexandra Wade, Timothy Olds, Ty Stanford, Hannah Keage, Montana Hunter, Nicholas Ware, Felicity M Simpson, Frini Karayanidis, Ashleigh E Smith

**Affiliations:** Alliance for Research in Exercise, Nutrition and Activity (ARENA), Allied Health and Human Performance, University of South Australia, Adelaide, South Australia, Australia; Alliance for Research in Exercise, Nutrition and Activity (ARENA), Allied Health and Human Performance, University of South Australia, Adelaide, South Australia, Australia; Alliance for Research in Exercise, Nutrition and Activity (ARENA), Allied Health and Human Performance, University of South Australia, Adelaide, South Australia, Australia; Alliance for Research in Exercise, Nutrition and Activity (ARENA), Allied Health and Human Performance, University of South Australia, Adelaide, South Australia, Australia; Murdoch Children’s Research Institute, Melbourne, Victoria, Australia; Alliance for Research in Exercise, Nutrition and Activity (ARENA), Allied Health and Human Performance, University of South Australia, Adelaide, South Australia, Australia; Behaviour-Brain-Body Research Centre, Justice and Society, University of South Australia, Adelaide, South Australia, Australia; School of Psychology and Vision Sciences, University of Leicester, Leicester, UK; Functional Neuroimaging Laboratory, School of Psychological Sciences, College of Engineering, Science and the Environment, University of Newcastle, Newcastle, New South Wales, Australia; Healthy Minds Research Program, Hunter Medical Research Institute, Newcastle, New South Wales, Australia; Functional Neuroimaging Laboratory, School of Psychological Sciences, College of Engineering, Science and the Environment, University of Newcastle, Newcastle, New South Wales, Australia; Healthy Minds Research Program, Hunter Medical Research Institute, Newcastle, New South Wales, Australia; Functional Neuroimaging Laboratory, School of Psychological Sciences, College of Engineering, Science and the Environment, University of Newcastle, Newcastle, New South Wales, Australia; Healthy Minds Research Program, Hunter Medical Research Institute, Newcastle, New South Wales, Australia; Alliance for Research in Exercise, Nutrition and Activity (ARENA), Allied Health and Human Performance, University of South Australia, Adelaide, South Australia, Australia

**Keywords:** Cognition, Physical activity, Sedentary behavior, Sleep, Time use

## Abstract

**Background:**

Each day is made up of a composition of “time-use behaviors.” These can be classified by their intensity (eg, light or moderate–vigorous physical activity [PA]) or domain (eg, chores, socializing). Intensity-based time-use behaviors are linked with cognitive function and cardiometabolic health in older adults, but it is unknown whether these relationships differ depending on the domain (or type/context) of behavior.

**Methods:**

This study included 397 older adults (65.5 ± 3.0 years, 69% female, 16.0 ± 3.0 years education) from Adelaide and Newcastle, Australia. Time-use behaviors were recorded using the Multimedia Activity Recall for Children and Adults, cognitive function was measured using the Addenbrooke’s Cognitive Examination III and Cambridge Neuropsychological Test Automated Battery, and systolic and diastolic blood pressure, total cholesterol, and waist–hip ratio were also recorded. Two 24-hour time-use compositions were derived from each participant’s Multimedia Activity Recall for Children and Adults, including a 4-part intensity composition (sleep, sedentary behavior, light, and moderate–vigorous PA) and an 8-part domain composition (Sleep, Self-Care, Chores, Screen Time, Quiet Time, Household Administration, Sport/Exercise, and Social).

**Results:**

Linear regressions found significant associations between the domain composition and both Addenbrooke’s Cognitive Examination III (*p* = .010) and waist–hip ratio (*p* = .009), and between the intensity composition and waist–hip ratio (*p* = .025). Isotemporal substitution modeling demonstrated that the domains of sedentary behaviors and PA impacted their associations with Addenbrooke’s Cognitive Examination III, while any PA appeared beneficial for waist–hip ratio.

**Conclusions:**

Findings suggest the domain of behavior should be considered when aiming to support cognitive function, whereas, for cardiometabolic health, it appears sufficient to promote any type of PA.

The relative distribution of physical activity, sedentary behavior, and sleep across the 24-hour day has important implications for cognitive and cardiometabolic health. The independent relationships between each of these “time-use behaviors” and cognitive and cardiometabolic outcomes have been investigated widely, but more recently, research has shifted toward understanding how the 24-hour composition of these behaviors (ie, balance of these behaviors across the 24-hour day) relates to health. A recent cross-sectional study using a compositional data analysis approach in over 15 000 adults (mean age 53.7 ± 9.7) demonstrated that spending more time in moderate–vigorous physical activity (MVPA) and limiting sedentary behavior (SB) was favorably associated with body mass index, waist circumference, and several blood-based markers of cardiovascular health (eg, high-density lipoprotein [HDL] cholesterol, triglycerides) (1). Similarly, a cross-sectional study in 1 411 adults by McGregor et al. (2) reported that spending more time in MVPA and less time in sedentary behavior was positively associated with body mass index, waist–hip ratio, and blood pressure, while another cross-sectional study by Farrahi et al. (3) also reported that spending less time sedentary and more time in active behaviors was associated with better cardiometabolic health (eg, adiposity, lipid biomarkers).

Despite growing evidence that 24-hour time-use composition is associated with cardiometabolic health, the findings for cognitive outcomes are less conclusive and evidence is sparse. Our recent review incorporated studies investigating how *combinations* of time-use behaviors (ie, 2 or more) are associated with cognitive function in older adults. We found that spending more time in higher-intensity physical activity (PA) (ie, moderate–vigorous PA; MVPA) and less time in sedentary behavior is positively related to cognitive function (4), which aligns broadly with evidence for cardiometabolic health. However, few studies have assessed all 24-hour behaviors together in the same model using a compositional approach, as has been done more frequently in cardiometabolic research (4–7). Thus far, there is insufficient evidence for a relationship between 24-hour time-use composition and cognitive function in older adults (see Falck et al. (8) for overview). This may be due to a number of lifestyle and sociodemographic factors that moderate the relationship between device-measured 24-hour time-use composition and cognitive function, including age, disability status, socioeconomic status, race, and ethnicity, as well as genetic predisposition to Alzheimer’s disease, which are often overlooked in this research area (6).

One additional consideration is that time-use behaviors are not one-dimensional, in that they can be quantified not only by their metabolic or intensity-based characteristics, but also by their contextual or behavioral characteristics. Somewhat antithetically to the “move more, sit less” notion for cardiometabolic health outcomes, some studies (albeit not using compositional approaches) have reported that higher engagement in sedentary behavior is positively associated with cognitive function (9). This is likely, at least in part, due to the types (or domains) of sedentary behaviors that participants engage in, and the level of cognitive engagement required of these tasks. For example, passive sedentary behaviors such as watching television have been associated with unfavorable cognitive outcomes in older adults (10), whereas cognitively stimulating activities such as reading, studying, or playing cards have been associated with better cognitive function (11).

To our knowledge, no previous studies have explored how the 24-hour composition of activity domains (rather than time spent in different intensity bands) relates to cognitive function in older adults. This is likely because many self-report time-use questionnaires do not comprehensively collect data on all daily behaviors. One self-report measure which captures activity and sleep patterns across 24 hours is the Multimedia Activity Recall for Children and Adults (MARCA) (12). Each activity recalled by the participant is linked to an activity compendium, allowing daily activities to be classified based on intensity (using metabolic equivalent cut points, eg, light VS. vigorous PA), or based on activity domains (eg, chores, recreational PA, screen time). The MARCA has been validated against accelerometry data in both children and adult samples (12,13). One previous study (14) used MARCA data to investigate how intensity-based and domain-based time-use compositions were differentially associated with respiratory and all-cause mortality risk in adults with chronic obstructive pulmonary disease, but to our knowledge, this approach (using the MARCA) has not yet been applied to cognitive or cardiometabolic outcomes in healthy older adults. Consequently, it is not well understood whether intensity-based and domain-based compositions are differentially associated with cardiometabolic health and cognitive function outcomes. To address this gap, this study explored the association between self-reported 24-hour activity patterns, cognitive function, and cardiometabolic health outcomes in healthy older adults, comparing 24-hour time-use compositions made up of intensity bands and activity domains.

## Method

### Ethics

The ACTIVate study (15) was prospectively registered with the Australian New Zealand Clinical Trials Registry (ACTRN12619001659190). Ethics approval was obtained from the University of South Australia and University of Newcastle Human Research Ethics Committee (202639). All procedures were conducted in accordance with the Declaration of Helsinki.

### Participants

The ACTIVate Study is a prospective longitudinal cohort study investigating associations between lifestyle behaviors and changes in cognition and health. Data used in the current study incorporate baseline assessments conducted between 2020 and 2021. The eligibility criteria and screening procedures for the ACTIVate study are described in detail elsewhere (15). Briefly, participants recruited from Adelaide and Newcastle, Australia, met inclusion criteria if they were aged 60–70 years, fluent in the English language, had no current clinical diagnosis of dementia, no major psychiatric or neurological diagnoses, and no known intellectual or major physical disability. Interested participants were required to complete a phone screening interview to assess eligibility based on these criteria, as well as undertaking the Montreal Cognitive Assessment (telephone version; T-MoCA) to screen for dementia using a cutoff score of <13/22.

### Study Measures

#### 24-hour activity patterns

A trained research assistant asked participants to self-report their activity and sleep patterns over the past 2 days using the MARCA. To begin the interview, participants were asked if (and when) they had eaten breakfast, lunch, or dinner on the day being recalled (ie, the day prior to the phone call). Starting at midnight, participants then recalled all activities they engaged in that day (ie, until midnight the following night) in 5-minute granularity for each activity, using meals as anchor points. This process was then repeated for the day prior to that (ie, 2 days before the phone call), resulting in two 24-hour recalls. Phone calls were conducted in the 7 days following initial study visits (where cognitive, cardiometabolic, and covariate measures were collected).

Each recalled activity was assigned to one of 520 discrete activities from an activity compendium (12,16). The information gathered for each activity included duration, metabolic equivalent (METs), and its hierarchical domain classification. In the traditional use of the MARCA, each activity is categorized into one of 9 “superdomains”: Sleep, Self-Care, Chores, Screen Time, Quiet Time, Transport, Social, Physical Activity, and Work/Study. Within each superdomain, activities are further classified by “macrodomain” (eg, within the “Quiet Time” superdomain, activities may fall under “Non-reading” (such as listening to music) or “Reading”) and “mesodomain” (eg, within the “Non-reading” macrodomain, activities may fall under “spiritual,” “sit,” or “listen to music,” among others). In line with a previous study (14), some adjustments were made to activity classifications for this study. Namely, activities which were originally classified as “Passive Transport” on the macrodomain level, or as “Work/Study” on the superdomain level, were combined to create a new superdomain “Household Administration.” Additionally, activities classified as “Active Transport” (mainly walking) on the macrodomain level, or as “Physical Activity” on the superdomain level, were combined to create a new superdomain “Sport/Exercise.” Thus, activities recalled within this study were classified under one of 8 mutually exclusive and exhaustive superdomains: Sleep, Self-Care, Chores, Screen Time, Quiet Time, Household Administration, Sport/Exercise, and Social. An exhaustive list of activities included in each superdomain, corresponding macrodomain and mesodomain classifications, and their MET values are provided in Supplementary Table 1.

#### Cognitive and cardiometabolic outcome measures

Global cognitive function was measured using the Addenbrooke’s Cognitive Examination III (ACE-III). The ACE-III is a paper-and-pencil-style cognitive screening tool which assesses 5 domains of cognitive function (memory, attention/orientation, language, fluency, and visuospatial ability). A total score out of 100 is generated which has demonstrated sensitivity to dementia using a cutoff score of 88/100 (17).

Domain-specific cognitive function was assessed using the Cambridge Neuropsychological Test Automated Battery (CANTAB). Participants completed a series of CANTAB tests which were then *z*-scored, reverse-scored (for tests where higher values represented poorer performance), and combined using taxonomy classifications (18) to create 3 cognitive composites: memory (Verbal Recognition Memory test), executive function (Multitasking and One Touch Stockings of Cambridge tests), and processing speed (Reaction Time test). A detailed description of methods used to create domain-specific cognitive composites can be found elsewhere (4).

Waist and hip circumferences (cm) were measured by a trained researcher according to the ISAK International Standards for Anthropometric Assessment (19). Using a measuring tape, waist circumference was measured at the narrowest point of the abdomen (or, at the midpoint between the lower costal border and iliac crest if narrowing was not obvious), while hip circumference was measured at the level of the greatest posterior protuberance of the buttocks. Both were measured twice, and the average waist and hip circumference were calculated. Finally, waist–hip ratio was calculated by taking the average waist circumference divided by the average hip circumference.

Clinic blood pressure (systolic and diastolic) was measured using an Omron blood pressure monitor with a blood pressure cuff fitted over the left brachial artery. Participants were seated for at least 5 minutes prior to the first blood pressure assessment. Three measurements were taken, 1 minute apart while participants were seated. Only the second and third measures were averaged to obtain the mean systolic and diastolic blood pressure values for this study.

Total cholesterol was derived from a fasting venous blood sample. Lipids were collected in a 9 mL ethylenediaminetraacetic acid (EDTA; 18 mg) anticoagulant vacuette tube (grenier bio-one, Kremsmünster, Austria). Samples were aliquoted after plasma separation at 4 000 rpm for 10 minutes and frozen at −80°C until analysis. At the time of analysis, samples were defrosted on ice before vortex and then centrifuged at 10 000 rpm for 2 minutes to remove particulates. Lipids were analyzed using the auto-analyzer KONELAB 20XTi (Thermo Fisher, Waltham, MA) with Thermo Fisher reagents. The instrument was calibrated with sCal (Thermo Fisher). One hundred and fifty microlitres of each sample was then added to a sample cup (taking care to remove bubbles) and placed into the auto-analyzer with the necessary reagents (Thermo Fisher) to be analyzed by testing for light absorbency.

#### Covariates

Demographic and lifestyle factors which have been identified as risk factors for dementia (both modifiable and non-modifiable) were captured for inclusion as potential covariates in analyses (20). Several factors were measured using self-report questionnaires, including age (years), sex (male, female), and hearing difficulties (no difficulties/difficulties with 2 or more people talking at the same time or in a noisy background/major hearing loss). Additional modifiable dementia risk factors were extracted from the Australian National University Alzheimer’s Disease Risk Index (21), including education (total years, including primary, secondary, and tertiary); frequency of alcohol consumption (“never,” “light-moderate,” “heavy”); smoking status (current, previous, never); history of head injury (yes/no); depression (Center for Epidemiological Studies—Depression scale (22), yes ≥16, no ≤15); and type 2 diabetes diagnosis (yes/no). For descriptive purposes only, self-reported retirement status (yes/no), Modified Monash Model classification (23) (rurality based on residential postcode reported at baseline), and subjective cognitive function (Cognitive Function Instrument [CFI] (24)) were also collected.

### Data Analysis

#### Creation of intensity- and domain-based time-use compositions

Analyses were conducted using R version 4.2 (R Core Team, 2023). Average daily time spent in intensity bands and superdomains (min/d) was explored descriptively using arithmetic means and standard deviations. Next, 2 time-use compositions were created for each participant. One 24-hour composition used time spent in intensity bands (min/d) to form 4 compositional parts: sleep (≤0.9 METs); sedentary behavior (>0.9–1.5 METs); light physical activity (≥1.5–2.9 METs); and moderate–vigorous physical activity (>2.9 METs), herein referred to as the ‘intensity composition.” The second 24-hour composition used the time spent in superdomains (min/d) to form 8 compositional parts: Sleep, Self-Care, Chores, Screen Time, Quiet Time, Household Administration, Sport/Exercise, and Social (herein referred to as the “domain composition”). Zero values in any compositional parts (*n* = 26 Quiet Time, *n* = 27 Screen Time, *n* = 35 Sport/Exercise, *n* = 4 Household Administration, *n* = 2 MVPA) were replaced using Expectation-Maximization algorithms implemented with the lrEM function (*zCompositions* package (25)). Average total daily time-use compositions (1437.5 ± 10.7 minutes) were closed to sum 1 440 minutes total by proportionally re-scaling all compositional parts, and were then expressed as a series of isometric log-ratio coordinates (*ilrs* (26)) to be included in linear regression models as predictors.

#### Model selection

To determine which covariates would be included in final analyses, Bayesian information criterion stepwise variable selection was applied (using *step* function in *stats* R package (27)). For each cognitive and cardiometabolic outcome and for both domain and intensity compositions (therefore, across 16 linear regression models), stepwise variable selection was performed, whereby the lower limit model required *ilrs* (ie, time-use composition) to be included, and the upper limit included *ilrs* and all potential covariates (age, sex, site, education, depression, hearing loss, diabetes, history of head injury, alcohol consumption, and smoking status). Models that elicited the minimum Bayesian information criterion value (and were therefore indicated to be the final model) were recorded, and commonalities across the 16 linear regression models were assessed (Supplementary Table 2). Following model selection, none of the final models included history of head injury, alcohol consumption, depression, or smoking status. For consistency across models, all analyses included age, sex, site, education, diabetes status, and hearing loss as covariates.

#### Statistical analysis

Multiple linear regression models were used to explore the associations between 24-hour time-use compositions (intensity or domain-based) and each outcome (ACE-III, memory, executive function, processing speed, waist–hip ratio, total cholesterol, systolic BP, and diastolic BP), using the lm() function in R. To check for nonlinear associations between ilrs and outcomes, an additional model was created whereby time-use composition was expressed as ilrs and quadratic terms (squared and interaction terms of the ilr sets). An *F*-test was used to determine whether the quadratic ilr terms were warranted in addition to the original model.

Variable significance (using alpha = 0.05) in models was determined using ANOVA *F* tests applied to linear models (*car* package (28)). To account for multiple comparisons, all *p* values from the final ANOVA *F-*test outputs were adjusted for false discovery rate (FDR) using the Benjamini–Hochberg method (29).

#### Modeling associations between reallocations of time and cognitive outcomes

To further explore any statistically significant relationships between 24-hour time-use composition and cognitive or cardiometabolic outcomes, compositional isotemporal substitution modeling was used to demonstrate how reallocations of time between time-use behaviors were associated with each outcome in the original compositional scale (30,31). Briefly, compositional isotemporal substitution is a method used to interpret regression coefficients from compositional multiple linear regression models. The method estimates the difference in an outcome (eg, cognitive performance or cardiometabolic health) when fixed durations of time are reallocated from one part of the composition (eg, sleep, in a 24-hour time-use composition) to another (eg, sedentary behavior), while the remaining parts (eg, LPA and MVPA) are kept constant (31). Reallocations are computed in reference to a starting composition (here, the mean time-use composition of the sample). Using these methods, we generated reallocations whereby predicted differences in outcomes were expressed in the original units of the outcome variable (eg, ACE-III scores ranging from 0–100). Reallocations were plotted using both proportional (eg, increasing time in sleep by 20 minutes while reallocating time to all other behaviors *pro-rata*) and one-for-one swaps (eg, increasing time in sleep by 20 minutes and taking that time directly from self-care). The latter modeling was performed to determine whether behavior change benefits were uniform or specific across activity types (eg, if increasing time in social activities was beneficial regardless of the activity it was reallocated to).

## Results

### Participant Demographics

Four hundred and twenty-six participants enrolled in the ACTIVate study at baseline. Of these, 425 participants completed the ACE-III, 422 had waist–hip ratio measurements, 419 had clinic blood pressure measurements, and 402 had cholesterol data. Of the 402 participants with complete clinic data, 397 had completed the 2-day MARCA recalls. Thus, the final sample included 397 older adults. Participants were aged 65.5 ± 3.0 years, were mostly female (69%) and retired (75%), and had 16.0 ± 3.0 years of education. Over half of the participants (54%) were from the Adelaide site, and across both sites, 88% of participants resided in metropolitan or inner regional areas (based on Modified Monash Model score of 1 or 2). On average, participants scored highly (95 ± 3.0, out of possible 100) on the ACE-III, and the mean score on the CFI (1.5 ± 1.6 out of a possible 14) indicated low levels of subjective cognitive complaints (Table 1).

**Table 1.** Descriptives of Final Sample

Variable	Level	Total
Age (years)		65.5 ± 3.0
Sex	Male	123 (31%)
	Female	273 (69%)
Education (years)		16.5 ± 3.2
Site	Adelaide	215 (54%)
	Newcastle	182 (46%)
Domain-based time-use behaviors (min/d)	Chores	206.9 ± 107.8
	Quiet time	87.4 ± 75.8
	Screen time	137.4 ± 86.7
	Self-care	127.7 ± 40.8
	Sleep	489.5 ± 65.3
	Social	142.0 ± 92.3
	Household administration	174.1 ± 124.9
	Sport/exercise	72.6 ± 65.2
Intensity-based time-use behaviors (min/d)	Sleep (<0.9 METs)	489.5 ± 65.3
	Sedentary behavior (0.9–1.5 METs)	531.8 ± 130.2
	Light PA (1.5–2.9 METs)	246.8 ± 89.1
	MVPA (>2.9 METs)	169.4 ± 96.6
Smoking status (*n* = 394)	Current	7 (2%)
	Previous	150 (38.0%)
	Never	237 (60.0%)
Alcohol consumption (*n* = 391)	Heavy	7 (1.8%)
	Light-moderate	38 (9.7%)
	None	346 (88.0%)
CES-D score ≥16 (depression) (*n* = 370)	Yes	27 (6.8%)
	No	343 (86%)
History of head injury (*n* = 388)	Yes	68 (17.0%)
	No	320 (81.0%)
Difficulty with hearing (*n* = 343)	Yes	139 (35.0%)
	No	214 (54.0%)
History of type II diabetes (*n* = 392)	Yes	19 (4.8%)
	No	373 (94.0%)
Waist to hip ratio (*n* = 393)		0.9 ± 0.1
Systolic BP (*n* = 387)		134.2 ± 18.4
Diastolic BP (*n* = 387)		80.8 ± 10.2
Total cholesterol (mmol/L) (*n* = 376)		5.5 ± 1.0
Retired (*n* = 396)		298 (75.1%)
MMM 2019 classification of 1 or 2 (metropolitan or inner regional)		352 (88%)
Addenbrooke’s Cognitive Examination III total score (*n* = 396)		95 ± 4
Scored ≤88 on ACE-III (*n* = 396)		19 (4.8%)
Cognitive Function Instrument total score (*n* = 396)		1.5 ± 1.6

*Notes*: Values are presented as mean ± *SD* for numeric variables, or count (percentage) for categorical variables. Domain and intensity components are presented as arithmetic means. Proportions for several variables do not total to 100% due to missing data. <88 ACE-III cutoff indicates possible dementia (17).

ACE-III = Addenbrooke’s Cognitive Examination III; BP = blood pressure; CES-D = Center for Epidemiological Studies Depression scale; MMM = Modified Monash Model; MVPA = moderate-vigorous physical activity.

**Figure 1.**
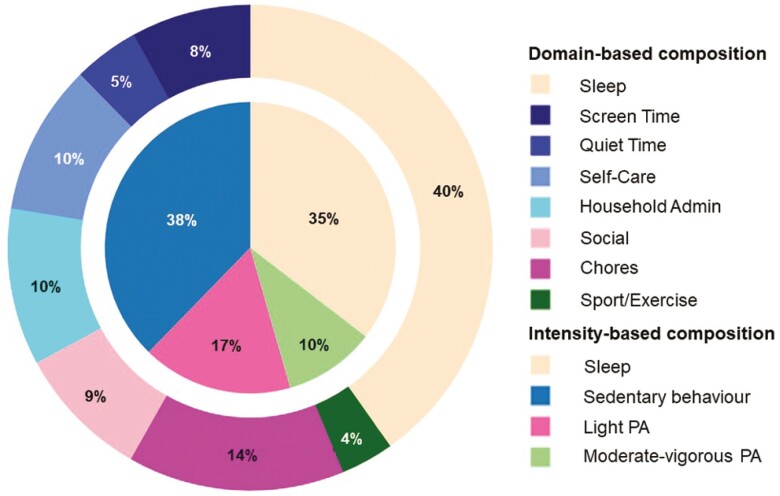
Intensity and domain compositions of the sample. The inner pie chart represents the 4-part intensity composition, while the outer donut chart represents the 8-part domain composition. Proportions represent the time-use composition under closure (ie, after compositions were rounded to 1440 minutes). Admin = administration; PA = physical activity.

Participants’ 24-hour time-use compositions are displayed in Figure 1 as both intensity (inner pie chart) and domain compositions (outer donut chart). Figure 1 represents time-use compositions after rounding to 1 440 minutes (ie, time-use compositions under closure). On average, according to the intensity composition, participants spent most of their day in sedentary behavior (9.0 hours), followed by sleep (8.5 hours), light PA (4.0 hours), and moderate–vigorous PA (2.4 hours). Domain compositions showed similar proportions, with participants spending most of their day in sleep (9.7 hours), followed by chores (3.5 hours), household administration (2.5 hours), self-care (2.4 hours), social activity (2.2 hours), screen time (1.9 hours), quiet time (1.0 hours), and sport/exercise (0.8 hours).

### Associations Between 24-Hour Time-Use Composition and Cognitive and Cardiometabolic Outcomes

Variable significance from the linear regression models are presented in Supplementary Tables 3 and 4. After adjustment for covariates and FDR, the domain composition was significantly associated with global cognition (*F* = 2.95, *p* = .010), but not the intensity composition (*F* = 1.17, *p* = .376). Conversely, both the domain (*F* = 3.20, *p* = .009) and intensity compositions (*F* = 4.03, *p* = .025) were significantly associated with waist–hip ratio after adjustment for covariates and FDR. Neither type of time-use composition was associated with the remaining cognitive and cardiometabolic outcomes including memory (domain: *F* = 2.25, *p* = .10; intensity: *F* = 2.22, *p* = .150), executive function (domain: *F* = 1.00, *p* = .598; intensity: *F* = 1.02, *p* = .535), processing speed (domain: *F* = 0.34, *p* = .937; intensity: *F* = 0.22, *p* = .883), total cholesterol (domain: *F* = 0.75, *p* = .689; intensity: *F* = 0.94, *p* = .582), systolic BP (domain: *F* = 1.23, *p* = .330; intensity: *F* = 0.61, *p* = .611), and diastolic BP (domain: *F* = 1.98, *p* = .080; intensity: *F* = 1.19, *p* = .441).

### Reallocations of Time, Cognitive Function, and Cardiometabolic Health Outcomes

To better understand the significant relationships between time-use compositions and ACE-III score (domain composition) and waist–hip ratio (both intensity and domain compositions), a series of proportional and one-for-one reallocations were generated, using the mean intensity and domain compositions as the reference composition. All one-for-one reallocations are displayed here: https://arena2024.shinyapps.io/context-intensity-app/.

#### Reallocations of time between domains and ACE-III score

**Figure 2.**
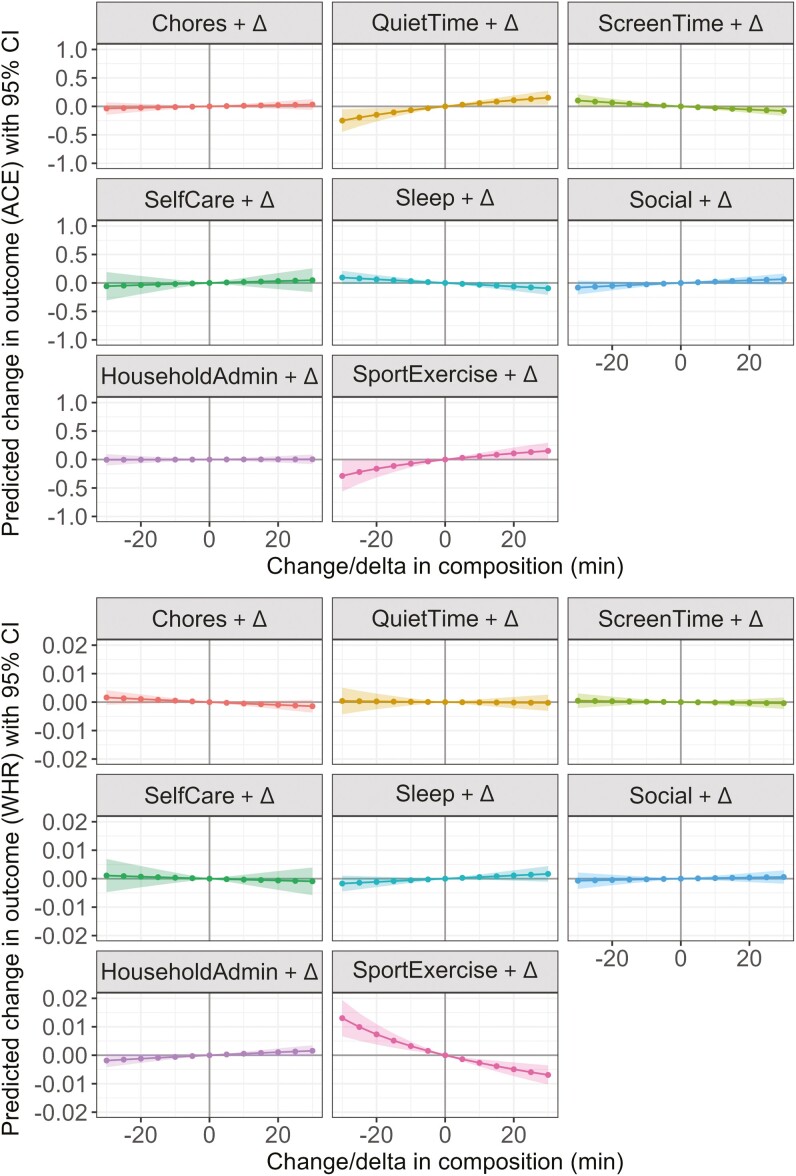
The model-predicted difference in Addenbrooke’s Cognitive Examination III score (Panel A, *y*-axis) and waist–hip ratio (Panel B, *y*-axis) associated with proportional reallocations of time toward or away from each superdomain (displayed in the header of each panel). Reallocations are based on increasing or decreasing time spent in each superdomain behavior from the mean domain composition, and range from −30 minutes (to the left of solid line) to +30 minutes (to the right of solid line) in 5-minute increments. Shading represents 95% confidence intervals. ACE = Addenbrooke’s Cognitive Examination III; WHR = waist–hip ratio.

More time spent in Quiet Time (such as reading and listening to music), Social and Sport/Exercise superdomains (at the equal expense of remaining behaviors) was associated with better ACE-III score, while more time in Sleep and Screen Time was associated with poorer ACE-III score (Figure 2, panel A). Only reallocations of time toward or away from Quiet Time and Sport/Exercise were statistically significant; however, the predicted differences in ACE-III score were less than the minimum possible difference (1 point). For example, increasing Quiet Time or Sport/Exercise by 30 minutes (while proportionally decreasing time in other behaviors) was associated with a predicted +0.15 difference in ACE-III score (Quiet Time 95% CI = 0.03, 0.27; Sport/Exercise 95% CI = 0.01, 0.30). One-for-one reallocations (across all time increments from +5 to +30 minutes) in a number of domains were associated with better ACE-III scores, including from Screen Time toward Sport/Exercise, Quiet Time or Social; from Sleep toward Quiet Time or Sport/Exercise; and from Household Administration toward Quiet Time. Despite being statistically significant, the predicted differences in ACE-III score were small, with the largest predicted difference seen for a 30-minute reallocation from Sport/Exercise to Screen Time (−0.35 ACE-III score, 95% CI = −0.63, −0.08).

#### Reallocations of time between domains and waist–hip ratio

More time spent in Sport/Exercise at the expense of the remaining domains (Figure 2, panel B) was associated with smaller waist–hip ratio, although the predicted magnitude of these differences was small (eg, +30 minutes Sport/Exercise = −0.007 waist–hip ratio, 95% CI = −0.01, −0.00). One-for-one reallocations confirmed that, regardless of which domain time was taken from (except for Self-Care) or the magnitude of the reallocation, more time in Sport/Exercise was significantly associated with smaller waist–hip ratio. Additionally, reallocating time from Household Administration (passive transport, work, and study) toward Chores was associated with smaller waist–hip ratio. A 30-minute reallocation from Sport/Exercise toward Household Administration was associated with the greatest (detrimental) difference in waist–hip ratio (+0.01, 95% CI = 0.00, 0.02).

#### Reallocations of time between intensity bands and waist–hip ratio

**Figure 3.**
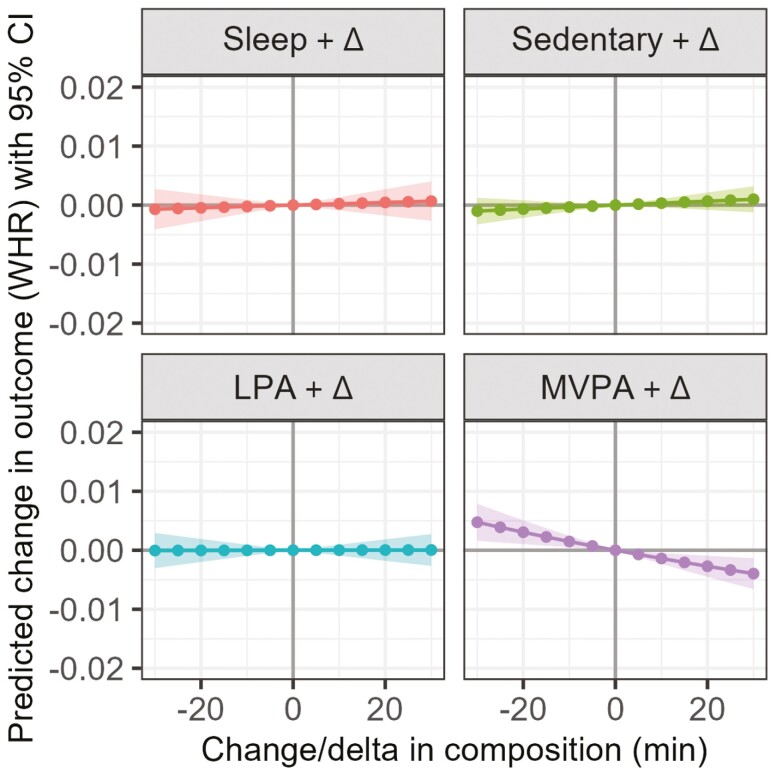
The model-predicted difference in waist–hip ratio (y-axis) associated with proportional reallocations of time toward or away from each intensity band (displayed in the header of each panel). Reallocations reflect increasing or decreasing time spent in each intensity band from the mean intensity composition, and range from −30 minutes (to the left of solid line) to +30 minutes (to the right of solid line) in 5-minute increments. Shading represents 95% confidence intervals. LPA = light-intensity physical activity; MVPA = moderate–vigorous physical activity; WHR = waist–hip ratio.

More time in MVPA, while proportionally decreasing time in LPA, SB, and sleep was significantly associated with waist–hip ratio, although the estimated differences were small (eg, +30 minutes of MVPA: −0.004 waist–hip ratio, 95% CI = −0.007, −0.001; Figure 3). One-for-one reallocations toward MVPA from LPA, sleep, or SB were associated with smaller waist–hip ratio. Out of the modeled reallocations, 30 minutes of SB at the expense of MVPA was associated with the largest (albeit detrimental) difference in waist–hip ratio (+0.005, 95% CI = 0.002, 0.007).

## Discussion

This study explored whether 24-hour time-use compositions consisting of either intensities (eg, sleep, SB, LPA, MVPA) or activity domains (eg, Sport/Exercise, Work, Screen Time) were differentially associated with cognitive performance (global cognition, memory, executive function, and processing speed) and a range of cardiometabolic health outcomes (waist–hip ratio, total cholesterol, systolic and diastolic blood pressure) in healthy older adults. We found that the domain composition but not the intensity composition was significantly associated with ACE-III score (global cognition), while both time-use compositions were significantly associated with waist–hip ratio (cardiometabolic outcome). We found no associations between either domain or intensity compositions and memory, executive function, processing speed, total cholesterol, systolic BP, or diastolic BP. Post-hoc compositional isotemporal substitution modeling showed that the domain composition had different associations with ACE-III than waist–hip ratio. For example, increasing time spent in Quiet Time (eg, reading, listening to music) at the expense of other predominantly sedentary domains like Screen Time (eg, watching TV) was beneficially associated with ACE-III score, while this reallocation was not associated with significant differences in waist–hip ratio. Conversely, reallocating time toward Sport/Exercise at the expense of almost any other domain (except for Self-Care) was associated with smaller waist–hip ratio, while this reallocation was only significantly associated with ACE-III score if time was taken from Screen Time or Sleep. Although the size of the predicted change in ACE-III and waist–hip ratio was small, the findings of this study raise several important points for consideration.

### The Context of Time Use Matters for Cognitive Function

Spending less time in sedentary behavior and more time in physical activity is favorably associated with cardiometabolic health outcomes such as adiposity, blood pressure, and cholesterol (1–3,32), but as highlighted by several recent reviews, evidence on the relationship between sedentary behavior and cognitive function is less conclusive (8,33). This is likely due, at least in part, to the types of sedentary behaviors that people engage in which may vary in terms of cognitive demand and context. Nuances in sedentary behaviors are not typically captured when considering total sedentary time as the exposure, and importantly, they may have contrasting flow-on effects on cognitive function (10,34,35). The findings of the current study support this notion in several ways. First, we found no evidence that total sedentary behavior (as captured in the intensity composition) was associated with ACE-III score, but there was an association with the domain composition which differentiated sedentary behaviors based on their superdomain classification. Second, we found that modeling increased time spent in some predominantly sedentary superdomains (Quiet Time, which may include reading, listening to music or some religious activities, or Social, which may include playing a musical instrument, sitting and talking with others, or crafts) predicted beneficial cognitive outcomes, while spending more time in the Screen Time superdomain (TV watching, video games) was detrimental. Several previous studies have reported similar negative relationships between more “cognitively passive” sedentary behaviors (eg, TV watching) and cognitive function (34,36) or dementia risk (37,38). In a large-scale study using the UK Biobank, Wu et al. (37) reported a hazard ratio of dementia of 1.28 for those who reported watching TV for ≥4 h/d compared to <1 h/d. Additionally, reallocating 30 min/d from TV watching to daily (habitual) physical activity or structured exercise was associated with a 6% and 12% lower dementia risk (HR 0.96 [95% CI 0.91, 0.97], and HR 0.88 [95% CI 0.83, 0.93], respectively) (37). Da Ronch et al. (36) reported that older adults who spent more time watching TV had worse performance on the Mini-Mental State Examination, while no such relationships were reported for other sedentary activities. Together, the current findings support the notion that not all sedentary behaviors are equal in their relationship with cognitive function. Instead, there is likely a hierarchy of how sedentary behaviors relate to cognitive function, in that some have positive effects and others have negative effects on cognitive function.

An additional finding of the current study was that increasing time in the Sport/Exercise superdomain at the expense of either Sleep or Screen Time was associated with better ACE-III score, while reallocating time to other domains involving predominantly physical activities (eg, Chores) was not. For example, reallocating 30 minutes from Screen Time to Sport/Exercise was associated with a statistically significant predicted difference in ACE-III score, while the same reallocation from Screen Time to Chores resulted in a positive but non-significant predicted difference (+0.24 vs +0.12). Despite the findings being exploratory and small in magnitude, they suggest that leisure-based or recreational physical activities may be more beneficial for general cognitive function compared to other domains such as household chores. Although a few previous studies have explored whether leisure-based PA and household PA are differentially associated with cognitive function, the findings are mixed (39,40). As the current literature is predominantly cross-sectional and varies greatly in both PA and cognitive measurement approaches, it is difficult to determine directionality or causality of the relationship (ie, perhaps greater cognitive function leads to higher engagement in certain physical activities).

Interestingly, we found no association between either the intensity or domain composition and memory, processing speed, or executive function outcomes, measured using a CANTAB. This finding aligns with a previous study in this cohort which found no association between device-based (wrist-worn accelerometry) measures of time use and the same cognitive outcomes (4). The lack of associations with these cognitive outcomes could be due to several factors, including limited variability in cognitive composite *z*-scores, or the theoretical framework (rather than data-driven factor analysis) used to generate the cognitive composites.

### Every Minute of Physical Activity Counts for Cardiometabolic Health

Spending more time in physical activity was favorably associated with waist–hip ratio. This was observed across both intensity composition models (ie, increasing time in MVPA at the expense of sleep, LPA, SB was associated with smaller waist–hip ratio) and domain composition models (ie, increasing time in Sport/Exercise or Chores superdomains at the expense of Screen Time, Sleep, Social, or Household Administration was associated with smaller waist–hip ratio). This suggests that physical activity is beneficial for adiposity regardless of intensity or type, although the strongest predicted associations were observed for higher-intensity activities (MVPA, or Sport/Exercise). Notably, the predicted impacts of reallocating time toward or away from MVPA or Sport/Exercise on waist–hip ratio were nonlinear, in that the predicted changes in waist–hip ratio associated with decreasing MVPA or Sport/Exercise were markedly stronger than with increasing these activities. Similar predicted relationships with waist–hip ratio were reported in a previous study by Dumuid et al. (41). Taken together, our findings align with evidence from a number of device-based studies that suggested substituting SB for MVPA is beneficial for cardiometabolic health (1,41–43). Additionally, our findings concur with a previous review of compositional data analysis studies, which concluded that the greatest benefits to cardiometabolic health were associated with reallocations of time toward MVPA (44).

Interestingly, we did not find any associations between time-use composition and other cardiometabolic health outcomes including systolic BP, diastolic BP, and total cholesterol. This aligns with a number of previous cross-sectional compositional data analysis studies. For example, Powell et al. (43) reported associations between time-use composition and adiposity measures, but no associations with total cholesterol or triglycerides. Similarly, Biddle et al. (42) reported that reallocating time from sitting to standing or stepping (ie, SB to LPA) was associated with lower waist circumference, as well as lower triglycerides and higher HDL cholesterol, but no associations with total cholesterol, low-density lipoprotein cholesterol, or hemoglobin A1c. Finally, Dumuid et al. (41) reported associations between time-use composition and measures of fitness and adiposity in a sample of older adults, but no associations with blood pressure (systolic or diastolic), total cholesterol, or blood glucose. Together, these findings suggest that spending greater proportions of the day in physically active behaviors has stronger benefits for body composition than for blood markers of cardiometabolic health. We speculate that this may be due to a number of factors. Firstly, it is well known that other lifestyle factors such as diet, smoking, and alcohol consumption have strong influences on cholesterol and blood pressure (45,46), so it is possible that these outcomes were less sensitive to activity and sleep patterns measured in this study. Secondly, directionality or causality cannot be inferred from cross-sectional data. This is important to consider in the context of cardiometabolic outcomes such as total cholesterol which are typically a reflection of cumulative lifestyle patterns over time, rather than current lifestyle patterns alone (47). Finally, daily time-use patterns and cardiometabolic outcomes such as blood pressure can fluctuate substantially across time, and it is possible that the single measurements taken were not truly representative of typical ranges for this cohort, thus impacting the observed relationships (48).

### Strengths and Limitations of the Study

A key strength of this study is that the interdependent and mutually exclusive nature of time-use behaviors in the 24-hour day was accounted for *via* the use of a compositional data analysis approach. The statistical significance of relationships between predictors and outcomes were only interpreted following adjustment for FDR, which reduces the likelihood of type I error (29). The MARCA time-use recall tool has been validated against objective measures including accelerometry and doubly labeled water (12,49) and is therefore considered a reliable self-report tool. Finally, this study employed model selection to ensure that modifiable dementia risk factors relevant to this cohort were included in the final models.

Converging with previous studies in the field, the use of a cross-sectional study design restricts the ability to make strong causal and directional conclusions about associations between time use, cognitive function, and cardiometabolic health. It is important to note that the ACTIVate Study participants were highly active, had high global cognition scores (with limited range), were highly educated, and were predominantly female (2:1) potentially limiting the generalizability of the findings. It is plausible that the associations between time-use composition and both cognitive and cardiometabolic outcomes would differ in a less active sample, a sample with shorter average sleep duration (ie, not meeting sleep guidelines), or a sample with a wider distribution of cognitive scores. Additionally, our domain composition grouped activities using the highest level of the classification hierarchy (superdomains). Thus, it is possible that some activities that were grouped within a superdomain may have differing relationships with the outcome that were not detected, and further, alternative activity compendia may have grouped activities differently, yielded differing and/or stronger associations to those observed using the current compendium.

### Future Directions

Our findings build the case for intervention strategies that incorporate both the unique contexts of daily activities alongside considering activity intensity for enhancing cognition. For example, to concurrently improve or maintain cognitive function *and* cardiometabolic health, it may be beneficial to tailor intervention strategies so that (1) higher-intensity physical activity levels are maintained or increased to sustain cardiometabolic health, and (2) passive sedentary behaviors such as screen time are substituted for either physical activity *or* sedentary behaviors which promote mental stimulation or social engagement. To facilitate the feasibility of interventions for older adult populations where increasing higher-intensity activities may not be appropriate, future studies should also explore whether equivalent cognitive benefits can be achieved by a number of behavioral change options to suit the preferences and abilities of participants (see Dumuid et al. (50), for example). There is a need for longitudinal studies to investigate whether changes in time spent within intensity bands or activity domains are associated with maintenance of, or changes in, cognitive function and cardiometabolic health. This should be measured using sensitive time-use recall tools which are able to collect information on both the intensity, type, and context of activities simultaneously.

## Conclusion

This cross-sectional compositional data analysis study found that 24-hour time-use compositions made up of activity domains were associated with cognitive function and adiposity, while compositions made up of activity intensity bands were only associated with adiposity. Post-hoc reallocation analyses suggested that the context of sedentary behaviors and physical activities impact their association with cognitive function, while any physical activity (regardless of context) appeared beneficial for adiposity.

## Supplementary Material

glae233_suppl_Supplementary_Tables_S1-S4
