# Supplementary material for: Should We Work Smarter or Harder for Our Health? A Comparison of Intensity and Domain-Based Time-Use Compositions and Their Associations With Cognitive and Cardiometabolic Health
Source: J Gerontol A Biol Sci Med Sci. 2024 Sep 19;79(11):glae233. doi: 10.1093/gerona/glae233 (PMC11512025; doi:10.1093/gerona/glae233)
Supplement: glae233_suppl_Supplementary_Tables_S1-S4 [file glae233_suppl_supplementary_tables_s1-s4.docx]

**Supplementary Material**

**Manuscript title:** Should we work smarter or harder for our health? A comparison of intensity and domain-based time-use compositions and their associations with cognitive and cardiometabolic health.

**Authors:** Maddison L Mellow, Dorothea Dumuid, Alexandra Wade, Timothy Olds, Ty Stanford, Hannah Keage, Montana Hunter, Nicholas Ware, Felicity M Simpson, Frini Karayanidis, Ashleigh E Smith.

**Table of Contents**

eTable 1 …………………………………………………………………………………………………………..2

eTable 2 ………………………………………………………………….............................................................22

eTable 3 ………………………………………………………………….............................................................23

eTable 4 ………………………………………………………………….............................................................25

# **eTable 1**

# Compendium of activities and their domain and intensity classifications

| **Intensity component** | **Code** | **Superdomain** | **Macrodomain** | **Mesodomain** | **METs** | **Activity** |
| --- | --- | --- | --- | --- | --- | --- |
| Sleep | 100010 | sleep | sleep | sleep | 0.9 | sleeping |
| SB | 221000 | household administration | passive transport | riding | 1.0 | riding in a car/truck |
|  | 221110 | household administration | passive transport | riding | 1.0 | riding in a train/tram/ferry |
|  | 221120 | household administration | passive transport | riding | 1.0 | riding in a bus |
|  | 221130 | household administration | passive transport | riding | 1.0 | riding in a plane |
|  | 115080 | household administration | study | study | 1.0 | writing - lying down |
|  | 110150 | quiet time | non-reading | lie awake | 1.0 | lying awake |
|  | 112020 | quiet time | non-reading | listen to music | 1.0 | listening to music/radio - lying |
|  | 120210 | quiet time | non-reading | quiet time nec | 1.0 | sitting in a spa |
|  | 120140 | quiet time | non-reading | sit | 1.0 | sitting quietly |
|  | 120190 | quiet time | non-reading | sit | 1.0 | meditating |
|  | 721150 | quiet time | non-reading | spiritual | 1.0 | praying |
|  | 113040 | quiet time | reading | reading | 1.0 | reading - lying down |
|  | 111030 | screen time | passive screen time | TV | 1.0 | watching TV - lying quietly |
|  | 121050 | screen time | passive screen time | TV | 1.0 | watching TV - sitting |
|  | 521060 | self-care | grooming and ablutions | grooming and ablutions | 1.0 | having hair/nails done by someone else |
| **Intensity component** | **Code** | **Superdomain** | **Macrodomain** | **Mesodomain** | **METs** | **Activity** |
|  | 521090 | self-care | grooming and ablutions | grooming and ablutions | 1.0 | sitting on toilet |
|  | 531100 | self-care | grooming and ablutions | grooming and ablutions | 1.0 | taking medicine |
|  | 114070 | social | communication | phone/SMS | 1.0 | talking on phone - lying down |
|  | 410200 | social | cultural | art | 1.0 | drawing - lying down |
|  | 121130 | social | socialising | social nec | 1.0 | sitting at the movies/cinema/theatre |
|  | 711240 | social | socialising | social nec | 1.0 | kissing/hugging |
|  | 114190 | social | communication | phone/SMS | 1.2 | sending text messages (SMS) - lying down |
|  | 130060 | social | socialising | social nec | 1.2 | standing quietly - eg standing in a line |
|  | 122160 | quiet time | non-reading | listen to music | 1.3 | listening to music/radio - sitting |
|  | 123110 | quiet time | reading | reading | 1.3 | reading - sitting |
|  | 622100 | chores | inside chores | inside chores nec | 1.5 | knitting or sewing |
|  | 820360 | household administration | employment | other occupations | 1.5 | lab household administration - sitting |
|  | 830120 | household administration | employment | primary | 1.5 | farming - milking with a machine |
|  | 820470 | household administration | employment | white collar | 1.5 | office household administration - sitting (eg filing/stapling/organisng documents) |
|  | 820350 | household administration | employment | white collar | 1.5 | sitting in a meeting |
|  | 820560 | household administration | employment | white collar | 1.5 | teaching - sitting and talking |
|  | 721160 | quiet time | non-reading | spiritual | 1.5 | sitting in church |
|  | 722190 | screen time | passive screen time | passive videogames | 1.5 | computer/console games (eg playstation/xbox/handheld) |
|  | 522030 | self-care | eating | eating | 1.5 | eating - sitting |
|  | 522130 | self-care | eating | eating | 1.5 | drinking - sitting |
|  | 521080 | self-care | grooming and ablutions | grooming and ablutions | 1.5 | sitting in bath |
|  | 124100 | social | communication | phone/SMS | 1.5 | talking on phone - sitting |
|  | 124170 | social | communication | phone/SMS | 1.5 | sending text messages (SMS) - sitting |
|  | 124090 | social | communication | talk | 1.5 | sitting - talking |
|  | 721010 | social | communication | talk | 1.5 | family get-togethers (eat talk sit relax) |
| **Intensity component** | **Code** | **Superdomain** | **Macrodomain** | **Mesodomain** | **METs** | **Activity** |
|  | 420000 | social | cultural | art | 1.5 | arts and crafts - sitting |
|  | 720180 | social | cultural | music | 1.5 | singing - sitting |
|  | 721240 | social | socialising | party | 1.5 | partying (mostly sitting) |
|  | 721220 | social | socialising | social nec | 1.5 | playing cards/puzzles/board games |
|  | 721230 | social | socialising | social nec | 1.5 | watching live sporting events |
|  | 712220 | social | socialising | social nec | 1.5 | casino gambling (eg pokies/roulette/blackjack) - sitting |
|  | 711250 | social | socialising | social nec | 1.5 | sexual activity |
|  | 721030 | social | socialising | social nec | 1.5 | amusement/theme park rides |
|  | 722170 | social | socialising | social nec | 1.5 | wrapping/unwrapping presents |
| LPA | 732201 | screen time | active screen time | active videogames | 1.7 | playing active video games (eg EyeToy/dance mat/arcade/Wii games) - light |
|  | 420050 | household administration | computer | computer | 1.8 | computer household administration (eg typing/internet) |
|  | 420030 | household administration | study | study | 1.8 | studying/homehousehold administration |
|  | 420040 | household administration | study | study | 1.8 | taking notes/class discussion |
|  | 420060 | household administration | study | study | 1.8 | writing - sitting |
|  | 430020 | quiet time | reading | reading | 1.8 | reading - standing |
|  | 134170 | social | communication | phone/SMS | 1.8 | talking on phone - standing |
|  | 134180 | social | communication | phone/SMS | 1.8 | sending text messages (SMS) - standing |
|  | 134120 | social | communication | talk | 1.8 | standing - talking |
|  | 430000 | social | cultural | art | 1.8 | arts and craft - standing |
|  | 420010 | social | cultural | art | 1.8 | drawing - sitting |
|  | 720030 | social | cultural | music | 1.8 | playing the accordeon |
|  | 731250 | social | socialising | party | 1.8 | partying (mostly standing) |
|  | 430080 | social | socialising | social nec | 1.8 | giving a talk/presentation |
|  | 342771 | sport/exercise | play | games | 1.9 | playing catch - light |
|  | 241161 | sport/exercise | active transport | walking | 1.9 | walking - pushing a pram - light |
|  | 721020 | chores | inside chores | child care | 2.0 | breast feeding |
|  | 631110 | chores | inside chores | cleaning house | 2.0 | making the bed |
|  | 630040 | chores | inside chores | food preparation | 2.0 | cooking - standing |
| **Intensity component** | **Code** | **Superdomain** | **Macrodomain** | **Mesodomain** | **METs** | **Activity** |
|  | 630370 | chores | inside chores | food preparation | 2.0 | making recess or lunch |
|  | 642080 | chores | inside chores | laundry | 2.0 | hanging out washing |
|  | 632050 | chores | inside chores | laundry | 2.0 | doing the laundry |
|  | 820630 | household administration | employment | white collar | 2.0 | ushering |
|  | 221140 | household administration | passive transport | driving | 2.0 | driving a car/light truck/bus |
|  | 532040 | self-care | eating | eating | 2.0 | eating while standing |
|  | 532140 | self-care | eating | eating | 2.0 | drinking while standing |
|  | 531010 | self-care | grooming and ablutions | grooming and ablutions | 2.0 | brushing teeth |
|  | 531070 | self-care | grooming and ablutions | grooming and ablutions | 2.0 | showering & towelling off |
|  | 531150 | self-care | grooming and ablutions | grooming and ablutions | 2.0 | shaving |
|  | 534110 | self-care | grooming and ablutions | grooming and ablutions | 2.0 | washing hands/face |
|  | 534120 | self-care | grooming and ablutions | grooming and ablutions | 2.0 | putting on makeup/sunscreen/contact lenses/grooming etc |
|  | 533020 | self-care | grooming and ablutions | grooming and ablutions | 2.0 | dressing & undressing |
|  | 533050 | self-care | grooming and ablutions | grooming and ablutions | 2.0 | getting ready for bed |
|  | 720040 | social | cultural | music | 2.0 | playing the cello |
|  | 720060 | social | cultural | music | 2.0 | playing the flute/recorder - sitting |
|  | 720070 | social | cultural | music | 2.0 | playing the guitar - sitting |
|  | 720090 | social | cultural | music | 2.0 | playing the horn |
|  | 720140 | social | cultural | music | 2.0 | playing woodwind instruments |
|  | 730180 | social | cultural | music | 2.0 | singing - standing |
|  | 341031 | sport/exercise | sport | gym | 2.0 | fitball exercises - light |
|  | 331491 | sport/exercise | sport | water sports | 2.0 | sailboard/windsurfing - light |
|  | 840550 | household administration | employment | other occupations | 2.2 | postman |
| **Intensity component** | **Code** | **Superdomain** | **Macrodomain** | **Mesodomain** | **METs** | **Activity** |
|  | 830280 | household administration | employment | white collar | 2.2 | librarian |
|  | 641130 | chores | inside chores | cleaning house | 2.3 | putting away clothes |
|  | 630270 | chores | inside chores | food preparation | 2.3 | washing or clearing dishes |
|  | 632500 | chores | inside chores | inside chores nec | 2.3 | putting up the christmas tree |
|  | 632090 | chores | inside chores | laundry | 2.3 | ironing |
|  | 642200 | chores | inside chores | shopping-related | 2.3 | shopping |
|  | 830050 | household administration | employment | white collar | 2.3 | using a photocopier - standing |
|  | 830380 | household administration | employment | white collar | 2.3 | filing - standing |
|  | 830520 | household administration | employment | white collar | 2.3 | checkout/fast food operator |
|  | 840370 | household administration | employment | white collar | 2.3 | bartending |
|  | 820450 | household administration | employment | white collar | 2.3 | tailoring - cutting/hand sewing/machine sewing |
|  | 830550 | household administration | employment | white collar | 2.3 | teaching - standing and talking |
|  | 135000 | household administration | study | study | 2.3 | writing - standing |
|  | 430100 | social | cultural | art | 2.3 | drawing - standing |
|  | 430070 | social | socialising | social nec | 2.3 | drama |
|  | 722230 | social | socialising | social nec | 2.3 | casino gambling (eg pokies/roulette/blackjack) - standing |
|  | 342191 | sport/exercise | play | games | 2.3 | frisbee (general) - light |
|  | 321941 | sport/exercise | sport | water sports | 2.3 | sailing/boating - light |
|  | 341601 | sport/exercise | sport | water sports | 2.3 | surfing (body or board) - light |
|  | 622030 | chores | inside chores | child care | 2.5 | child care - sitting (eg feeding/bathing) |
|  | 641060 | chores | inside chores | cleaning house | 2.5 | dusting |
|  | 631490 | chores | inside chores | cleaning house | 2.5 | cleaning the bathroom/toilet |
|  | 640190 | chores | inside chores | food preparation | 2.5 | serving food |
|  | 640350 | chores | inside chores | food preparation | 2.5 | setting the table |
|  | 642360 | chores | inside chores | inside chores nec | 2.5 | fixing things up (eg bike/toys/mechanical household administration on car etc) |
| **Intensity component** | **Code** | **Superdomain** | **Macrodomain** | **Mesodomain** | **METs** | **Activity** |
|  | 632380 | chores | inside chores | inside chores nec | 2.5 | building a fire in a fireplace |
|  | 642070 | chores | inside chores | inside chores nec | 2.5 | feeding or caring for pets/farm animals (eg brushing) |
|  | 430140 | chores | inside chores | inside chores nec | 2.5 | packing/unpacking bag |
|  | 642170 | chores | inside chores | shopping-related | 2.5 | putting away or carrying groceries |
|  | 643460 | chores | outside chores | garden | 2.5 | fertilising/seeding/watering lawn |
|  | 643280 | chores | outside chores | garden | 2.5 | watering plants |
|  | 632390 | chores | outside chores | non-garden | 2.5 | filling car with petrol/gas |
|  | 830320 | household administration | employment | blue collar | 2.5 | operating heavy duty equipment (not driving) |
|  | 830330 | household administration | employment | other occupations | 2.5 | policing - directing traffic - standing |
|  | 820620 | household administration | employment | primary | 2.5 | mowing lawn with ride on mower |
|  | 241090 | household administration | passive transport | driving | 2.5 | riding a motorcycle or motor scooter |
|  | 820110 | household administration | passive transport | driving | 2.5 | driving a tractor/harvester |
|  | 531000 | self-care | grooming and ablutions | grooming and ablutions | 2.5 | brushing or combing hair (standing) |
|  | 730120 | social | cultural | music | 2.5 | playing the trumpet |
|  | 720100 | social | cultural | music | 2.5 | playing the piano or organ |
|  | 720130 | social | cultural | music | 2.5 | playing the violin |
|  | 721000 | social | socialising | social nec | 2.5 | camping - sitting |
|  | 321950 | sport/exercise | play | animals | 2.5 | playing with animals - sitting |
|  | 342772 | sport/exercise | play | games | 2.5 | playing catch - medium |
|  | 332040 | sport/exercise | play | games | 2.5 | pool/billiards/snooker |
|  | 731210 | sport/exercise | play | games | 2.5 | darts |
|  | 331590 | sport/exercise | sport | gym | 2.5 | stretching exercises |
|  | 331630 | sport/exercise | sport | gym | 2.5 | tai chi/yoga |
|  | 341110 | sport/exercise | sport | partner sports | 2.5 | croquet |
|  | 321870 | sport/exercise | sport | water sports | 2.5 | fishing - sitting |
|  | 241162 | sport/exercise | active transport | walking | 2.5 | walking - pushing a pram - medium |
| **Intensity component** | **Code** | **Superdomain** | **Macrodomain** | **Mesodomain** | **METs** | **Activity** |
|  | 820300 | sport/exercise | sport | miscellaneous sports | 2.6 | horse racing - walking |
|  | 741530 | social | Maori activities | Maori activities | 2.7 | Maori activities - Haka Tutungurahu (female) |
|  | 341032 | sport/exercise | sport | gym | 2.7 | fitball exercises - medium |
|  | 342761 | chores | inside chores | child care | 2.8 | playing with young children - light |
|  | 341291 | sport/exercise | play | animals | 2.8 | playing with animals - walk/run - light |
| MVPA | 632020 | chores | inside chores | child care | 3.0 | child care - standing (eg carrying/dressing/playing) |
|  | 642010 | chores | inside chores | child care | 3.0 | carrying small children |
|  | 641230 | chores | inside chores | cleaning house | 3.0 | tidying/cleaning room |
|  | 641370 | chores | inside chores | cleaning house | 3.0 | taking out the rubbish/garbage bin |
|  | 642400 | chores | inside chores | inside chores nec | 3.0 | getting ready to leave (eg close windows/shut/lock doors) |
|  | 642510 | chores | inside chores | inside chores nec | 3.0 | loading/unloading car |
|  | 633340 | chores | outside chores | garden | 3.0 | picking flowers fruit or vegetables |
|  | 642260 | chores | outside chores | non-garden | 3.0 | washing car or windows |
|  | 840100 | household administration | employment | blue collar | 3.0 | buffing the floor |
|  | 830400 | household administration | employment | blue collar | 3.0 | assembly line household administration |
|  | 820270 | household administration | employment | blue collar | 3.0 | machinery fitter |
|  | 830350 | household administration | employment | blue collar | 3.0 | toolmaker |
|  | 830010 | household administration | employment | blue collar | 3.0 | fixing the car - general |
|  | 840390 | household administration | employment | other occupations | 3.0 | nursing - patient care |
|  | 840480 | household administration | employment | other occupations | 3.0 | setting up chairs/light furniture for an event |
|  | 820130 | household administration | employment | primary | 3.0 | farming - milking by hand |
|  | 830600 | household administration | employment | white collar | 3.0 | stacking shelves |
|  | 840580 | household administration | employment | white collar | 3.0 | waitressing |
| **Intensity component** | **Code** | **Superdomain** | **Macrodomain** | **Mesodomain** | **METs** | **Activity** |
|  | 840590 | household administration | employment | white collar | 3.0 | door to door sales/delivery - long periods of walking |
|  | 221150 | household administration | passive transport | driving | 3.0 | driving a heavy truck (eg semi trailer) |
|  | 730080 | social | cultural | music | 3.0 | playing the guitar - standing |
|  | 331420 | sport/exercise | play | games | 3.0 | quoits |
|  | 341231 | sport/exercise | play | games | 3.0 | hacky sack - light |
|  | 341970 | sport/exercise | play | games | 3.0 | mini golf or putt putt |
|  | 342192 | sport/exercise | play | games | 3.0 | frisbee (general) - medium |
|  | 342621 | sport/exercise | play | games | 3.0 | table tennis - light |
|  | 342060 | sport/exercise | play | games | 3.0 | tenpin bowling |
|  | 331961 | sport/exercise | sport | gym | 3.0 | lifting weights - light |
|  | 341221 | sport/exercise | sport | gym | 3.0 | gymnastics - light |
|  | 321291 | sport/exercise | sport | miscellaneous sports | 3.0 | horseback riding - light |
|  | 342121 | sport/exercise | sport | snow sports | 3.0 | curling - light |
|  | 342370 | sport/exercise | sport | team sports | 3.0 | lawn bowls |
|  | 342691 | sport/exercise | sport | team sports | 3.0 | volleyball (court) - light |
|  | 341671 | sport/exercise | sport | track and field | 3.0 | athletics (track and field): throwing - light |
|  | 331492 | sport/exercise | sport | water sports | 3.0 | sailboard/windsurfing - medium |
|  | 321942 | sport/exercise | sport | water sports | 3.0 | sailing/boating - medium |
|  | 341091 | sport/exercise | sport | water sports | 3.0 | rowing/canoeing - light |
|  | 341602 | sport/exercise | sport | water sports | 3.0 | surfing (body or board) - medium |
|  | 341931 | sport/exercise | sport | water sports | 3.0 | swimming (playing in pool) - light |
|  | 342001 | sport/exercise | sport | water sports | 3.0 | water aerobics - light |
|  | 331870 | sport/exercise | sport | water sports | 3.0 | fishing - standing |
|  | 331880 | sport/exercise | sport | water sports | 3.0 | fishing - in the water with waders |
|  | 240051 | sport/exercise | active transport | walking | 3.0 | walking - light |
|  | 241171 | sport/exercise | active transport | walking | 3.0 | walking - pushing a wheelchair - light |
|  | 741260 | social | socialising | party | 3.2 | partying (mostly dancing) |
|  | 342773 | sport/exercise | play | games | 3.2 | playing catch - hard |
|  | 241163 | sport/exercise | active transport | walking | 3.2 | walking - pushing a pram - hard |
|  | 641210 | chores | inside chores | cleaning house | 3.3 | sweeping floor |
| **Intensity component** | **Code** | **Superdomain** | **Macrodomain** | **Mesodomain** | **METs** | **Activity** |
|  | 830090 | household administration | employment | white collar | 3.3 | bakery household administration |
|  | 341251 | sport/exercise | active transport | new locomotions | 3.3 | riding a scooter - light |
|  | 622040 | chores | inside chores | inside chores nec | 3.4 | sewing with machine |
|  | 732202 | screen time | active screen time | active videogames | 3.4 | playing active video games (eg EyeToy/dance mat/arcade/Wii games) - medium |
|  | 341033 | sport/exercise | sport | gym | 3.4 | fitball exercises - hard |
|  | 341211 | sport/exercise | sport | miscellaneous sports | 3.4 | golf - light |
|  | 342011 | sport/exercise | sport | partner sports | 3.4 | badminton - light |
|  | 641120 | chores | inside chores | cleaning house | 3.5 | mopping |
|  | 641250 | chores | inside chores | cleaning house | 3.5 | vacuuming |
|  | 631240 | chores | inside chores | inside chores nec | 3.5 | unpacking boxes |
|  | 632000 | chores | inside chores | inside chores nec | 3.5 | bathing dog |
|  | 633480 | chores | outside chores | garden | 3.5 | using garden power tools (eg power cutter/leaf blower/edger/snow blower) |
|  | 840030 | household administration | employment | blue collar | 3.5 | wiring/plumbing |
|  | 830260 | household administration | employment | other occupations | 3.5 | locksmith |
|  | 830290 | household administration | employment | white collar | 3.5 | petrol station attendant |
|  | 321010 | household administration | passive transport | driving | 3.5 | snowmobiling |
|  | 730110 | social | cultural | music | 3.5 | playing the trombone |
|  | 341011 | sport/exercise | sport | gym | 3.5 | gym - stationary rowing - light |
|  | 341870 | sport/exercise | sport | gym | 3.5 | light home exercises (eg back exercises) |
|  | 331000 | sport/exercise | sport | miscellaneous sports | 3.5 | archery |
|  | 741590 | social | Maori activities | Maori activities | 3.6 | Maori activities - Whakaeke (female) |
|  | 341791 | sport/exercise | sport | dance | 3.6 | ballet - light |
|  | 341801 | sport/exercise | sport | dance | 3.6 | tap/jazz dancing - light |
|  | 741580 | social | Maori activities | Maori activities | 3.7 | Maori activities - Waiata Tira (female) |
|  | 641180 | chores | inside chores | cleaning house | 3.8 | scrubbing floors in bathroom or bathtub |
| **Intensity component** | **Code** | **Superdomain** | **Macrodomain** | **Mesodomain** | **METs** | **Activity** |
|  | 741720 | social | Maori activities | Maori activities | 3.8 | Maori activities - Whakawhiti (female) |
|  | 331991 | sport/exercise | play | games | 3.8 | totem tennis - light |
|  | 342193 | sport/exercise | play | games | 3.8 | frisbee (general) - hard |
|  | 341021 | sport/exercise | sport | gym | 3.8 | gym - elliptical trainer - light |
|  | 342021 | sport/exercise | sport | team sports | 3.8 | baseball - light |
|  | 342101 | sport/exercise | sport | team sports | 3.8 | cricket - light |
|  | 342561 | sport/exercise | sport | team sports | 3.8 | softball or t-ball - light |
|  | 321943 | sport/exercise | sport | water sports | 3.8 | sailing/boating - hard |
|  | 341341 | sport/exercise | sport | water sports | 3.8 | kayaking - light |
|  | 341551 | sport/exercise | sport | water sports | 3.8 | snorkeling - light |
|  | 341603 | sport/exercise | sport | water sports | 3.8 | surfing (body or board) - hard |
|  | 341741 | sport/exercise | sport | water sports | 3.8 | whitewater rafting - light |
|  | 341271 | sport/exercise | active transport | new locomotions | 3.8 | riding a skateboard - light |
|  | 240052 | sport/exercise | active transport | walking | 3.8 | walking - medium |
|  | 741540 | social | Maori activities | Maori activities | 3.9 | Maori activities - Kapahaka (female) |
|  | 741550 | social | Maori activities | Maori activities | 3.9 | Maori activities - Moteatea (female) |
|  | 342762 | chores | inside chores | child care | 4.0 | playing with young children - medium |
|  | 643330 | chores | outside chores | garden | 4.0 | gardening |
|  | 440160 | chores | outside chores | non-garden | 4.0 | carrying sports equipment |
|  | 643220 | chores | outside chores | non-garden | 4.0 | sweeping outside |
|  | 830000 | household administration | employment | blue collar | 4.0 | fixing the car - body household administration |
|  | 830340 | household administration | employment | other occupations | 4.0 | policing - making an arrest - standing |
|  | 830310 | household administration | employment | other occupations | 4.0 | masseuse - giving massages - standing |
|  | 830530 | household administration | employment | white collar | 4.0 | teaching PE (not participating in class) |
|  | 720050 | social | cultural | music | 4.0 | playing the drums |
|  | 741570 | social | Maori activities | Maori activities | 4.0 | Maori activities - Waiata-a-ringa (female) |
|  | 341292 | sport/exercise | play | animals | 4.0 | playing with animals - walk/run - medium |
|  | 341232 | sport/exercise | play | games | 4.0 | hacky sack - medium |
|  | 342622 | sport/exercise | play | games | 4.0 | table tennis - medium |
| **Intensity component** | **Code** | **Superdomain** | **Macrodomain** | **Mesodomain** | **METs** | **Activity** |
|  | 331330 | sport/exercise | play | games | 4.0 | juggling |
|  | 331962 | sport/exercise | sport | gym | 4.0 | lifting weights - medium |
|  | 341222 | sport/exercise | sport | gym | 4.0 | gymnastics - medium |
|  | 321292 | sport/exercise | sport | miscellaneous sports | 4.0 | horseback riding - medium |
|  | 341041 | sport/exercise | sport | snow sports | 4.0 | snowboarding - light |
|  | 342122 | sport/exercise | sport | snow sports | 4.0 | curling - medium |
|  | 342040 | sport/exercise | sport | team sports | 4.0 | coaching (eg football/soccer/basketball) |
|  | 342692 | sport/exercise | sport | team sports | 4.0 | volleyball (court) - medium |
|  | 341672 | sport/exercise | sport | track and field | 4.0 | athletics (track and field): throwing - medium |
|  | 341932 | sport/exercise | sport | water sports | 4.0 | swimming (playing in pool) - medium |
|  | 342002 | sport/exercise | sport | water sports | 4.0 | water aerobics - medium |
|  | 241172 | sport/exercise | active transport | walking | 4.0 | walking - pushing a wheelchair - medium |
|  | 840560 | household administration | employment | other occupations | 4.1 | garbage collector |
|  | 643310 | chores | outside chores | garden | 4.3 | raking leaves |
|  | 840140 | household administration | employment | primary | 4.3 | farming - tending to cattle (eg feeding/hauling water) |
|  | 830200 | household administration | employment | primary | 4.3 | forestry - weeding/using a power saw |
|  | 341252 | sport/exercise | active transport | new locomotions | 4.4 | riding a scooter - medium |
|  | 632420 | chores | inside chores | inside chores nec | 4.5 | painting - inside the house |
|  | 623320 | chores | outside chores | garden | 4.5 | pulling up weeds |
|  | 830061 | household administration | employment | blue collar | 4.5 | carpentry - light |
|  | 840040 | household administration | employment | blue collar | 4.5 | sanding floors with power sander |
|  | 342401 | sport/exercise | play | games | 4.5 | paddleball - light |
|  | 341331 | sport/exercise | sport | gym | 4.5 | using a punching bag - light |
|  | 341212 | sport/exercise | sport | miscellaneous sports | 4.5 | golf - medium |
|  | 342012 | sport/exercise | sport | partner sports | 4.5 | badminton - medium |
|  | 341651 | sport/exercise | sport | track and field | 4.5 | athletics (track and field): jumping - light |
| **Intensity component** | **Code** | **Superdomain** | **Macrodomain** | **Mesodomain** | **METs** | **Activity** |
|  | 331731 | sport/exercise | sport | water sports | 4.5 | water skiing - light |
|  | 240091 | sport/exercise | active transport | walking | 4.5 | walking carrying a load - light |
|  | 741510 | social | Maori activities | Maori activities | 4.6 | Maori activities - Poi |
|  | 741710 | social | Maori activities | Maori activities | 4.7 | Maori activities - Whakawatea (female) |
|  | 341792 | sport/exercise | sport | dance | 4.8 | ballet - medium |
|  | 341802 | sport/exercise | sport | dance | 4.8 | tap/jazz dancing - medium |
|  | 341951 | sport/exercise | sport | gym | 4.8 | gym - push ups/sit ups/pull ups etc - light |
|  | 341131 | sport/exercise | sport | dance | 4.9 | dancing (general) - light |
|  | 341891 | sport/exercise | sport | track and field | 4.9 | race walking - light |
|  | 342763 | chores | inside chores | child care | 5.0 | playing with young children - hard |
|  | 643300 | chores | outside chores | garden | 5.0 | wheelbarrowing |
|  | 633390 | chores | outside chores | garden | 5.0 | shoveling/digging |
|  | 643440 | chores | outside chores | non-garden | 5.0 | carrying/loading/stacking wood |
|  | 632430 | chores | outside chores | non-garden | 5.0 | cleaning the gutters |
|  | 632410 | chores | outside chores | non-garden | 5.0 | painting - outside the house |
|  | 741450 | social | Maori activities | Maori activities | 5.0 | Maori activities - Moteatea (male) |
|  | 741560 | social | Maori activities | Maori activities | 5.0 | Maori activities - Taiaha (female) |
|  | 341293 | sport/exercise | play | animals | 5.0 | playing with animals - walk/run - hard |
|  | 331992 | sport/exercise | play | games | 5.0 | totem tennis - medium |
|  | 341233 | sport/exercise | play | games | 5.0 | hacky sack - hard |
|  | 342623 | sport/exercise | play | games | 5.0 | table tennis - hard |
|  | 331963 | sport/exercise | sport | gym | 5.0 | lifting weights - hard |
|  | 341751 | sport/exercise | sport | gym | 5.0 | aerobics/health hustle - light |
|  | 341223 | sport/exercise | sport | gym | 5.0 | gymnastics - hard |
|  | 321293 | sport/exercise | sport | miscellaneous sports | 5.0 | horseback riding - hard |
|  | 341531 | sport/exercise | sport | snow sports | 5.0 | skiing (downhill) - light |
|  | 342123 | sport/exercise | sport | snow sports | 5.0 | curling - hard |
|  | 342022 | sport/exercise | sport | team sports | 5.0 | baseball - medium |
|  | 342102 | sport/exercise | sport | team sports | 5.0 | cricket - medium |
|  | 342562 | sport/exercise | sport | team sports | 5.0 | softball or t-ball - medium |
|  | 342693 | sport/exercise | sport | team sports | 5.0 | volleyball (court) - hard |
| **Intensity component** | **Code** | **Superdomain** | **Macrodomain** | **Mesodomain** | **METs** | **Activity** |
|  | 341673 | sport/exercise | sport | track and field | 5.0 | athletics (track and field): throwing - hard |
|  | 331493 | sport/exercise | sport | water sports | 5.0 | sailboard/windsurfing - hard |
|  | 341342 | sport/exercise | sport | water sports | 5.0 | kayaking - medium |
|  | 341552 | sport/exercise | sport | water sports | 5.0 | snorkeling - medium |
|  | 341742 | sport/exercise | sport | water sports | 5.0 | whitewater rafting - medium |
|  | 341933 | sport/exercise | sport | water sports | 5.0 | swimming (playing in pool) - hard |
|  | 342003 | sport/exercise | sport | water sports | 5.0 | water aerobics - hard |
|  | 341272 | sport/exercise | active transport | new locomotions | 5.0 | riding a skateboard - medium |
|  | 240053 | sport/exercise | active transport | walking | 5.0 | walking - hard |
|  | 240071 | sport/exercise | active transport | walking | 5.0 | climbing stairs - light |
|  | 240092 | sport/exercise | active transport | walking | 5.0 | walking carrying a load - medium |
|  | 241080 | sport/exercise | active transport | walking | 5.0 | walking using crutches |
|  | 241173 | sport/exercise | active transport | walking | 5.0 | walking - pushing a wheelchair - hard |
|  | 341022 | sport/exercise | sport | gym | 5.1 | gym - elliptical trainer - medium |
|  | 830410 | household administration | employment | blue collar | 5.3 | steel mill - fettling/forging/tipping moulds |
|  | 741520 | social | Maori activities | Maori activities | 5.3 | Maori activities - Haka Powhiri (female) |
|  | 341911 | sport/exercise | sport | gym | 5.3 | gym - ski machine - light |
|  | 342431 | sport/exercise | sport | partner sports | 5.3 | racquetball - light |
|  | 342641 | sport/exercise | sport | partner sports | 5.3 | tennis (court) - light |
|  | 341042 | sport/exercise | sport | snow sports | 5.3 | snowboarding - medium |
|  | 342051 | sport/exercise | sport | snow sports | 5.3 | bobsled toboggan luge - light |
|  | 342181 | sport/exercise | sport | team sports | 5.3 | soccer (field/indoor) - light |
|  | 341461 | sport/exercise | active transport | new locomotions | 5.3 | rollerskating - light |
|  | 830210 | household administration | employment | primary | 5.4 | forestry - hoeing/slow axe chopping/planting by hand) |
|  | 741480 | social | Maori activities | Maori activities | 5.4 | Maori activities - Waiata Tira (male) |
|  | 643290 | chores | outside chores | garden | 5.5 | mowing lawn |
|  | 840490 | household administration | employment | blue collar | 5.5 | construction household administration outside |
|  | 830150 | household administration | employment | primary | 5.5 | farming - shovelling grain |
|  | 341941 | sport/exercise | sport | gym | 5.5 | gym - stationary cycling/bike - light |
| **Intensity component** | **Code** | **Superdomain** | **Macrodomain** | **Mesodomain** | **METs** | **Activity** |
|  | 341301 | sport/exercise | sport | snow sports | 5.5 | ice skating - light |
|  | 341253 | sport/exercise | active transport | new locomotions | 5.5 | riding a scooter - hard |
|  | 341213 | sport/exercise | sport | miscellaneous sports | 5.6 | golf - hard |
|  | 342013 | sport/exercise | sport | partner sports | 5.6 | badminton - hard |
|  | 741470 | social | Maori activities | Maori activities | 5.9 | Maori activities - Waiata-a-ringa (male) |
|  | 641410 | chores | inside chores | inside chores nec | 6.0 | carrying very heavy items (eg moving furniture) |
|  | 633400 | chores | outside chores | non-garden | 6.0 | chopping wood |
|  | 633450 | chores | outside chores | non-garden | 6.0 | using a chainsaw |
|  | 632470 | chores | outside chores | non-garden | 6.0 | shovelling snow |
|  | 830460 | household administration | employment | blue collar | 6.0 | using heavy power tools (eg pneumatic drill/jackhammer) |
|  | 830062 | household administration | employment | blue collar | 6.0 | carpentry - medium |
|  | 840020 | household administration | employment | blue collar | 6.0 | roofing |
|  | 830270 | household administration | employment | primary | 6.0 | horse grooming |
|  | 830610 | household administration | employment | primary | 6.0 | butchering animals |
|  | 830511 | household administration | employment | primary | 6.0 | digging ditches - light |
|  | 830160 | household administration | employment | primary | 6.0 | farming - grooming/brushing/shearing farm animals |
|  | 342402 | sport/exercise | play | games | 6.0 | paddleball - medium |
|  | 342201 | sport/exercise | play | games | 6.0 | frisbee (ultimate) - light |
|  | 341793 | sport/exercise | sport | dance | 6.0 | ballet - hard |
|  | 341803 | sport/exercise | sport | dance | 6.0 | tap/jazz dancing - hard |
|  | 341332 | sport/exercise | sport | gym | 6.0 | using a punching bag - medium |
|  | 341451 | sport/exercise | sport | miscellaneous sports | 6.0 | rockclimbing - light |
|  | 341532 | sport/exercise | sport | snow sports | 6.0 | skiing (downhill) - medium |
|  | 342271 | sport/exercise | sport | snow sports | 6.0 | hockey (ice) - light |
|  | 342031 | sport/exercise | sport | team sports | 6.0 | basketball - light |
| **Intensity component** | **Code** | **Superdomain** | **Macrodomain** | **Mesodomain** | **METs** | **Activity** |
|  | 342151 | sport/exercise | sport | team sports | 6.0 | football (Australian/Gaelic/American) - light |
|  | 342251 | sport/exercise | sport | team sports | 6.0 | European handball (team) - light |
|  | 342261 | sport/exercise | sport | team sports | 6.0 | hockey (field) - light |
|  | 342361 | sport/exercise | sport | team sports | 6.0 | lacrosse - light |
|  | 342381 | sport/exercise | sport | team sports | 6.0 | netball - light |
|  | 342701 | sport/exercise | sport | team sports | 6.0 | volleyball (beach) - light |
|  | 342811 | sport/exercise | sport | team sports | 6.0 | touch football - light |
|  | 341652 | sport/exercise | sport | track and field | 6.0 | athletics (track and field): jumping - medium |
|  | 331732 | sport/exercise | sport | water sports | 6.0 | water skiing - medium |
|  | 341241 | sport/exercise | active transport | cycling | 6.0 | riding a bicycle/bike - light |
|  | 240072 | sport/exercise | active transport | walking | 6.0 | climbing stairs - medium |
|  | 741440 | social | Maori activities | Maori activities | 6.2 | Maori activities - Kapahaka (male) |
|  | 741490 | social | Maori activities | Maori activities | 6.3 | Maori activities - Whakaeke (male) |
|  | 331993 | sport/exercise | play | games | 6.3 | totem tennis - hard |
|  | 342023 | sport/exercise | sport | team sports | 6.3 | baseball - hard |
|  | 342103 | sport/exercise | sport | team sports | 6.3 | cricket - hard |
|  | 342563 | sport/exercise | sport | team sports | 6.3 | softball or t-ball - hard |
|  | 341343 | sport/exercise | sport | water sports | 6.3 | kayaking - hard |
|  | 341553 | sport/exercise | sport | water sports | 6.3 | snorkeling - hard |
|  | 341743 | sport/exercise | sport | water sports | 6.3 | whitewater rafting - hard |
|  | 341273 | sport/exercise | active transport | new locomotions | 6.3 | riding a skateboard - hard |
|  | 732203 | screen time | active screen time | active videogames | 6.4 | playing active video games (eg EyeToy/dance mat/arcade/Wii games) - hard |
|  | 341023 | sport/exercise | sport | gym | 6.4 | gym - elliptical trainer - hard |
|  | 341952 | sport/exercise | sport | gym | 6.4 | gym - push ups/sit ups/pull ups etc - medium |
|  | 341051 | sport/exercise | sport | snow sports | 6.4 | snowshoeing - light |
|  | 830070 | household administration | employment | blue collar | 6.5 | coal mining (eg drilling coal/rock/erecting supports) |
|  | 830440 | household administration | employment | blue collar | 6.5 | loading/unloading truck |
|  | 840540 | household administration | employment | white collar | 6.5 | teaching PE (participating in class) |
|  | 741430 | social | Maori activities | Maori activities | 6.5 | Maori activities - Haka Tutungurahu (male) |
| **Intensity component** | **Code** | **Superdomain** | **Macrodomain** | **Mesodomain** | **METs** | **Activity** |
|  | 341132 | sport/exercise | sport | dance | 6.5 | dancing (general) - medium |
|  | 820290 | sport/exercise | sport | miscellaneous sports | 6.5 | horse racing - trotting |
|  | 341892 | sport/exercise | sport | track and field | 6.5 | race walking - medium |
|  | 240093 | sport/exercise | active transport | walking | 6.5 | walking carrying a load - hard |
|  | 341043 | sport/exercise | sport | snow sports | 6.6 | snowboarding - hard |
|  | 741610 | social | Maori activities | Maori activities | 6.7 | Maori activities - Whakawatea (male) |
|  | 341921 | sport/exercise | sport | gym | 6.8 | gym - stair machine - light |
|  | 341391 | sport/exercise | sport | track and field | 6.8 | orienteering - light |
|  | 830500 | household administration | employment | blue collar | 7.0 | masonry - building with bricks |
|  | 830080 | household administration | employment | blue collar | 7.0 | coal mining (eg shoveling coal) |
|  | 830512 | household administration | employment | primary | 7.0 | digging ditches - medium |
|  | 830220 | household administration | employment | primary | 7.0 | forestry - sawing by hand/barking trees/stacking |
|  | 741620 | social | Maori activities | Maori activities | 7.0 | Maori activities - Whakawhiti (male) |
|  | 341012 | sport/exercise | sport | gym | 7.0 | gym - stationary rowing - medium |
|  | 341942 | sport/exercise | sport | gym | 7.0 | gym - stationary cycling/bike - medium |
|  | 341912 | sport/exercise | sport | gym | 7.0 | gym - ski machine - medium |
|  | 341752 | sport/exercise | sport | gym | 7.0 | aerobics/health hustle - medium |
|  | 342432 | sport/exercise | sport | partner sports | 7.0 | racquetball - medium |
|  | 342642 | sport/exercise | sport | partner sports | 7.0 | tennis (court) - medium |
|  | 341302 | sport/exercise | sport | snow sports | 7.0 | ice skating - medium |
|  | 341521 | sport/exercise | sport | snow sports | 7.0 | skiing (cross-country) - light |
|  | 341571 | sport/exercise | sport | snow sports | 7.0 | speed skating (competitive) - light |
|  | 342052 | sport/exercise | sport | snow sports | 7.0 | bobsled toboggan luge - medium |
|  | 342182 | sport/exercise | sport | team sports | 7.0 | soccer (field/indoor) - medium |
|  | 331010 | sport/exercise | sport | water sports | 7.0 | jetskiing |
|  | 341092 | sport/exercise | sport | water sports | 7.0 | rowing/canoeing - medium |
|  | 341541 | sport/exercise | sport | water sports | 7.0 | skindiving (SCUBA) - light |
|  | 341611 | sport/exercise | sport | water sports | 7.0 | swimming laps - light |
| **Intensity component** | **Code** | **Superdomain** | **Macrodomain** | **Mesodomain** | **METs** | **Activity** |
|  | 341462 | sport/exercise | active transport | new locomotions | 7.0 | rollerskating - medium |
|  | 741410 | social | Maori activities | Maori activities | 7.1 | Maori activities - Haka |
|  | 741460 | social | Maori activities | Maori activities | 7.1 | Maori activities - Taiaha (male) |
|  | 830063 | household administration | employment | blue collar | 7.5 | carpentry - hard |
|  | 342403 | sport/exercise | play | games | 7.5 | paddleball - hard |
|  | 341321 | sport/exercise | sport | gym | 7.5 | karate/martial arts/judo/kick boxing - light |
|  | 341333 | sport/exercise | sport | gym | 7.5 | using a punching bag - hard |
|  | 342161 | sport/exercise | sport | team sports | 7.5 | rugby league - light |
|  | 342171 | sport/exercise | sport | team sports | 7.5 | rugby union - light |
|  | 341653 | sport/exercise | sport | track and field | 7.5 | athletics (track and field): jumping - hard |
|  | 341661 | sport/exercise | sport | track and field | 7.5 | athletics (track and field): hurdles steeplechase - light |
|  | 331733 | sport/exercise | sport | water sports | 7.5 | water skiing - hard |
|  | 342081 | sport/exercise | sport | water sports | 7.5 | water polo - light |
|  | 830420 | household administration | employment | blue collar | 7.9 | steel mill - tending furnace/hand rolling/merchant mill rolling |
|  | 840190 | household administration | employment | other occupations | 8.0 | fire fighting - hauling hoses on the ground |
|  | 840570 | household administration | employment | other occupations | 8.0 | climbing ladder |
|  | 830230 | household administration | employment | primary | 8.0 | forestry - felling trees |
|  | 830170 | household administration | employment | primary | 8.0 | farming - forking straw/bailing hay/cleaning barn |
|  | 342202 | sport/exercise | play | games | 8.0 | frisbee (ultimate) - medium |
|  | 341953 | sport/exercise | sport | gym | 8.0 | gym - push ups/sit ups/pull ups etc - hard |
|  | 341960 | sport/exercise | sport | gym | 8.0 | gym - circuit training with minimal rest |
|  | 341471 | sport/exercise | sport | gym | 8.0 | skipping/jump rope - light |
|  | 341452 | sport/exercise | sport | miscellaneous sports | 8.0 | rockclimbing - medium |
|  | 820280 | sport/exercise | sport | miscellaneous sports | 8.0 | horse racing - galloping |
|  | 341522 | sport/exercise | sport | snow sports | 8.0 | skiing (cross-country) - medium |
|  | 341533 | sport/exercise | sport | snow sports | 8.0 | skiing (downhill) - hard |
| **Intensity component** | **Code** | **Superdomain** | **Macrodomain** | **Mesodomain** | **METs** | **Activity** |
|  | 342272 | sport/exercise | sport | snow sports | 8.0 | hockey (ice) - medium |
|  | 342032 | sport/exercise | sport | team sports | 8.0 | basketball - medium |
|  | 342152 | sport/exercise | sport | team sports | 8.0 | football (Australian/Gaelic/American) - medium |
|  | 342252 | sport/exercise | sport | team sports | 8.0 | European handball (team) - medium |
|  | 342262 | sport/exercise | sport | team sports | 8.0 | hockey (field) - medium |
|  | 342362 | sport/exercise | sport | team sports | 8.0 | lacrosse - medium |
|  | 342382 | sport/exercise | sport | team sports | 8.0 | netball - medium |
|  | 342702 | sport/exercise | sport | team sports | 8.0 | volleyball (beach) - medium |
|  | 342812 | sport/exercise | sport | team sports | 8.0 | touch football - medium |
|  | 341481 | sport/exercise | sport | track and field | 8.0 | running/jogging - light |
|  | 341242 | sport/exercise | active transport | cycling | 8.0 | riding a bicycle/bike - medium |
|  | 240073 | sport/exercise | active transport | walking | 8.0 | climbing stairs - hard |
|  | 341133 | sport/exercise | sport | dance | 8.1 | dancing (general) - hard |
|  | 341893 | sport/exercise | sport | track and field | 8.1 | race walking - hard |
|  | 741420 | social | Maori activities | Maori activities | 8.5 | Maori activities - Haka Powhiri (male) |
|  | 341013 | sport/exercise | sport | gym | 8.5 | gym - stationary rowing - hard |
|  | 341753 | sport/exercise | sport | gym | 8.5 | aerobics/health hustle - hard |
|  | 341052 | sport/exercise | sport | snow sports | 8.5 | snowshoeing - medium |
|  | 341943 | sport/exercise | sport | gym | 8.8 | gym - stationary cycling/bike - hard |
|  | 341913 | sport/exercise | sport | gym | 8.8 | gym - ski machine - hard |
|  | 342433 | sport/exercise | sport | partner sports | 8.8 | racquetball - hard |
|  | 342643 | sport/exercise | sport | partner sports | 8.8 | tennis (court) - hard |
|  | 342053 | sport/exercise | sport | snow sports | 8.8 | bobsled toboggan luge - hard |
|  | 342183 | sport/exercise | sport | team sports | 8.8 | soccer (field/indoor) - hard |
|  | 341463 | sport/exercise | active transport | new locomotions | 8.8 | rollerskating - hard |
|  | 830513 | household administration | employment | primary | 9.0 | digging ditches - hard |
|  | 830240 | household administration | employment | primary | 9.0 | forestry - trimming trees |
|  | 341922 | sport/exercise | sport | gym | 9.0 | gym - stair machine - medium |
|  | 342581 | sport/exercise | sport | partner sports | 9.0 | squash - light |
|  | 341303 | sport/exercise | sport | snow sports | 9.0 | ice skating - hard |
| **Intensity component** | **Code** | **Superdomain** | **Macrodomain** | **Mesodomain** | **METs** | **Activity** |
|  | 341523 | sport/exercise | sport | snow sports | 9.0 | skiing (cross-country) - hard |
|  | 341572 | sport/exercise | sport | snow sports | 9.0 | speed skating (competitive) - medium |
|  | 341392 | sport/exercise | sport | track and field | 9.0 | orienteering - medium |
|  | 341311 | sport/exercise | active transport | new locomotions | 9.4 | rollerblading (in-line skating) - light |
|  | 342203 | sport/exercise | play | games | 10.0 | frisbee (ultimate) - hard |
|  | 341472 | sport/exercise | sport | gym | 10.0 | skipping/jump rope - medium |
|  | 341322 | sport/exercise | sport | gym | 10.0 | karate/martial arts/judo/kick boxing - medium |
|  | 342273 | sport/exercise | sport | snow sports | 10.0 | hockey (ice) - hard |
|  | 342033 | sport/exercise | sport | team sports | 10.0 | basketball - hard |
|  | 342153 | sport/exercise | sport | team sports | 10.0 | football (Australian/Gaelic/American) - hard |
|  | 342162 | sport/exercise | sport | team sports | 10.0 | rugby league - medium |
|  | 342172 | sport/exercise | sport | team sports | 10.0 | rugby union - medium |
|  | 342253 | sport/exercise | sport | team sports | 10.0 | European handball (team) - hard |
|  | 342263 | sport/exercise | sport | team sports | 10.0 | hockey (field) - hard |
|  | 342363 | sport/exercise | sport | team sports | 10.0 | lacrosse - hard |
|  | 342703 | sport/exercise | sport | team sports | 10.0 | volleyball (beach) - hard |
|  | 342813 | sport/exercise | sport | team sports | 10.0 | touch football - hard |
|  | 341662 | sport/exercise | sport | track and field | 10.0 | athletics (track and field): hurdles steeplechase - medium |
|  | 341482 | sport/exercise | sport | track and field | 10.0 | running/jogging - medium |
|  | 341612 | sport/exercise | sport | water sports | 10.0 | swimming laps - medium |
|  | 342082 | sport/exercise | sport | water sports | 10.0 | water polo - medium |
|  | 341243 | sport/exercise | active transport | cycling | 10.0 | riding a bicycle/bike - hard |
|  | 342383 | sport/exercise | sport | team sports | 10.1 | netball - hard |
|  | 341053 | sport/exercise | sport | snow sports | 10.6 | snowshoeing - hard |
|  | 830430 | household administration | employment | blue collar | 11.0 | steel mill - removing slag |
|  | 840180 | household administration | employment | other occupations | 11.0 | fire fighting - climbing ladder with full gear |
|  | 341453 | sport/exercise | sport | miscellaneous sports | 11.0 | rockclimbing - hard |
|  | 341613 | sport/exercise | sport | water sports | 11.0 | swimming laps - hard |
|  | 341923 | sport/exercise | sport | gym | 11.3 | gym - stair machine - hard |
|  | 341393 | sport/exercise | sport | track and field | 11.3 | orienteering - hard |
| **Intensity component** | **Code** | **Superdomain** | **Macrodomain** | **Mesodomain** | **METs** | **Activity** |
|  | 341473 | sport/exercise | sport | gym | 12.0 | skipping/jump rope - hard |
|  | 342582 | sport/exercise | sport | partner sports | 12.0 | squash - medium |
|  | 341093 | sport/exercise | sport | water sports | 12.0 | rowing/canoeing - hard |
|  | 341323 | sport/exercise | sport | gym | 12.5 | karate/martial arts/judo/kick boxing - hard |
|  | 342163 | sport/exercise | sport | team sports | 12.5 | rugby league - hard |
|  | 342173 | sport/exercise | sport | team sports | 12.5 | rugby union - hard |
|  | 341663 | sport/exercise | sport | track and field | 12.5 | athletics (track and field): hurdles steeplechase - hard |
|  | 341483 | sport/exercise | sport | track and field | 12.5 | running/jogging - hard |
|  | 341542 | sport/exercise | sport | water sports | 12.5 | skindiving (SCUBA) - medium |
|  | 342083 | sport/exercise | sport | water sports | 12.5 | water polo - hard |
|  | 341312 | sport/exercise | active transport | new locomotions | 12.5 | rollerblading (in-line skating) - medium |
|  | 342583 | sport/exercise | sport | partner sports | 15.0 | squash - hard |
|  | 341573 | sport/exercise | sport | snow sports | 15.0 | speed skating (competitive) - hard |
|  | 341313 | sport/exercise | active transport | new locomotions | 15.6 | rollerblading (in-line skating) - hard |
|  | 341543 | sport/exercise | sport | water sports | 16.0 | skindiving (SCUBA) - hard |
|  | 830250 | household administration | employment | primary | 17.0 | forestry - axe chopping (fast) |

*Note.* ‘Intensity component’ and ‘Superdomain’ columns reflect the classification of each activity (in the far-right column) in their intensity and domain compositions, respectively. METs = metabolic equivalents.

# **eTable 2**

# Outcomes of model selection

| **Model** | ilrs | Age | Sex | Site | Education | Depression | Hearing | Diabetes | TBI | Alcohol | Smoking |
| --- | --- | --- | --- | --- | --- | --- | --- | --- | --- | --- | --- |
| ACE ~ domain-based *ilrs* | **●** | **×** | **×** | **●** | **●** | **×** | **●** | **×** | **×** | **×** | **×** |
| ACE ~ intensity-based *ilrs* | **●** | **×** | **×** | **●** | **●** | **×** | **●** | **×** | **×** | **×** | **×** |
| Memory ~ domain-based *ilrs* | **●** | **×** | **×** | **●** | **×** | **×** | **×** | **×** | **×** | **×** | **×** |
| Memory ~ intensity-based *ilrs* | **●** | **×** | **×** | **●** | **×** | **×** | **×** | **×** | **×** | **×** | **×** |
| Processing speed ~ domain-based *ilrs* | **●** | **●** | **×** | **×** | **×** | **×** | **×** | **×** | **×** | **×** | **×** |
| Processing speed ~ intensity-based *ilrs* | **●** | **●** | **×** | **×** | **×** | **×** | **×** | **×** | **×** | **×** | **×** |
| Executive function ~ domain-based *ilrs* | **●** | **●** | **●** | **×** | **×** | **×** | **×** | **×** | **×** | **×** | **×** |
| Executive function ~ intensity-based *ilrs* | **●** | **●** | **●** | **×** | **×** | **×** | **×** | **×** | **×** | **×** | **×** |
| Waist:hip ratio ~ domain-based *ilrs* | **●** | **×** | **●** | **×** | **×** | **×** | **×** | **×** | **×** | **×** | **×** |
| Waist:hip ratio ~ intensity-based *ilrs* | **●** | **×** | **●** | **×** | **×** | **×** | **×** | **×** | **×** | **×** | **×** |
| Total cholesterol ~ domain-based *ilrs* | **●** | **×** | **●** | **×** | **×** | **×** | **×** | **●** | **×** | **×** | **×** |
| Total cholesterol ~ intensity-based *ilrs* | **●** | **×** | **●** | **×** | **×** | **×** | **×** | **●** | **×** | **×** | **×** |
| SBP ~ domain-based *ilrs* | **●** | **●** | **●** | **●** | **●** | **×** | **×** | **×** | **×** | **×** | **×** |
| SBP ~ intensity-based *ilrs* | **●** | **●** | **●** | **●** | **●** | **×** | **×** | **×** | **×** | **×** | **×** |
| DBP ~ domain-based *ilrs* | **●** | **×** | **●** | **●** | **×** | **×** | **×** | **●** | **×** | **×** | **×** |
| DBP ~ intensity-based *ilrs* | **●** | **×** | **●** | **●** | **●** | **×** | **×** | **●** | **×** | **×** | **×** |

*Note.* Green circles (‘**●**’) indicates covariates that were included in final models based on Bayesian information criterion. Grey crosses (‘**×**’) indicate covariates that were not included in final models. ACE = Addenbrooke’s Cognitive Examination III; SBP = systolic blood pressure; DBP = diastolic blood pressure

**eTable 3**

Statistical output of ANOVA Type II F-test for cognitive outcomes

|  |  | **Global cognition** | | | **Memory** | | | **Executive function** | | | **Processing speed** | | |
| --- | --- | --- | --- | --- | --- | --- | --- | --- | --- | --- | --- | --- | --- |
|  |  | F (n,d) | *p* | *adj.p* | F (n,d) | *p* | *adj.p* | F (n,d) | *p* | *adj.p* | F (n,d) | *p* | *adj.p* |
| **Domain model** | *ilrs* | 2.95  (7, 336) | 0.005 | **0.010** | 2.25  (7, 322) | 0.030 | 0.104 | 1.00  (7, 324) | 0.427 | 0.598 | 0.34  (7, 328) | 0.937 | 0.937 |
|  | Age | 0.00  (1, 336) | 0.953 | 0.953 | 0.31  (1, 322) | 0.572 | 0.668 | 18.30  (1, 324) | <0.001 | **<0.001** | 9.09  (1, 328) | 0.003 | **0.019** |
|  | Sex | 0.27  (1, 336) | 0.607 | 0.709 | 2.49  (1, 322) | 0.115 | 0.202 | 9.72  (1, 324) | 0.002 | **0.007** | 1.46  (1, 328) | 0.228 | 0.399 |
|  | Site | 16.63  (1, 336) | <0.001 | **<0.001** | 4.86  (1, 322) | 0.028 | 0.104 | 2.90  (1, 324) | 0.090 | 0.209 | 3.54  (1, 328) | 0.061 | 0.212 |
|  | Education | 15.29  (1, 336) | <0.001 | **<0.001** | 3.96  (1, 322) | 0.047 | 0.110 | 2.04  (1, 324) | 0.154 | 0.269 | 0.03  (1, 328) | 0.855 | 0.937 |
|  | Diabetes | 1.47  (1, 336) | 0.227 | 0.318 | 0.06  (1, 322) | 0.813 | 0.813 | 0.09  (1, 324) | 0.768 | 0.768 | 1.70  (1, 328) | 0.193 | 0.399 |
|  | Hearing loss | 7.72  (1, 336) | 0.006 | **0.010** | 1.85  (1, 322) | 0.175 | 0.245 | 0.15  (1, 324) | 0.699 | 0.768 | 0.03  (1, 328) | 0.869 | 0.937 |
| **Intensity model** | *ilrs* | 1.17  (3, 340) | 0.323 | 0.376 | 2.22  (3, 326) | 0.086 | 0.150 | 1.02  (3, 328) | 0.382 | 0.535 | 0.22  (3, 332) | 0.883 | 0.883 |
|  | Age | 0.01  (1, 340) | 0.906 | 0.906 | 0.36  (1, 326) | 0.547 | 0.638 | 18.01  (1, 328) | <0.001 | **<0.001** | 9.94  (1, 332) | 0.002 | **0.012** |
|  | Sex | 1.53  (1, 340) | 0.217 | 0.376 | 3.01  (1, 326) | 0.084 | 0.150 | 9.87  (1, 328) | 0.002 | **0.007** | 2.37  (1, 332) | 0.124 | 0.299 |
|  | Site | 17.86  (1, 340) | <0.001 | **<0.001** | 5.27  (1, 326) | 0.022 | 0.094 | 2.59  (1, 328) | 0.109 | 0.225 | 4.08  (1, 332) | 0.044 | 0.155 |
|  | Education | 18.85  (1, 340) | <0.001 | **<0.001** | 4.94  (1, 326) | 0.027 | 0.094 | 2.32  (1, 328) | 0.129 | **0.891** | 0.06  (1, 332) | 0.809 | 0.883 |
|  | Diabetes | 1.04  (1, 340) | 0.308 | 0.376 | 0.01  (1, 326) | 0.919 | 0.919 | 0.02  (1, 328) | 0.891 | 0.891 | 1.87  (1, 332) | 0.172 | 0.300 |
|  | Hearing loss | 6.34  (1, 340) | 0.012 | **0.028** | 2.08  (1, 326) | 0.150 | 0.210 | 0.14  (1, 328) | 0.713 | 0.832 | 0.05  (1, 332) | 0.824 | 0.883 |

*Note.* ANOVA F test outcomes of final models (covariate adjusted) for cognitive outcomes. ilrs= isometric log-ratios used to express 24-hour time-use behaviours as a composition; ‘Domain model’ = models whereby time-use composition was created using activity superdomains as compositional parts. Bold text and asterisks indicate p-values which remained statistically significant (p<0.05) following false discovery rate adjustment. F(n,d) = F statistic, and numerator and denominator degrees of freedom.

**eTable 4**

Statistical output of ANOVA Type II F-test for cardiometabolic outcomes

|  |  | **Waist:hip ratio** | | | **Total cholesterol** | | | **Systolic BP** | | | **Diastolic BP** | | |
| --- | --- | --- | --- | --- | --- | --- | --- | --- | --- | --- | --- | --- | --- |
|  |  | F (n,d) | *p* | *adj.p* | F (n,d) | *p* | *adj.p* | F (n,d) | *p* | *adj.p* | F (n,d) | *p* | *adj.p* |
| **Domain model** | *ilrs* | 3.20  (7, 335) | 0.003 | 0.009 | 0.75  (7, 319) | 0.634 | 0.689 | 1.23  (7, 332) | 0.284 | 0.330 | 1.98  (7, 332) | 0.057 | 0.080 |
|  | Age | 0.70  (1, 335) | 0.403 | 0.471 | 0.16  (1, 319) | 0.689 | 0.689 | 7.81  (1, 332) | 0.005 | **0.013** | 0.12  (1, 332) | 0.729 | 0.729 |
|  | Sex | 141.25  (1, 335) | <0.001 | **<0.001** | 15.46  (1, 319) | <0.001 | **<0.001** | 20.65  (1, 332) | <0.001 | **<0.001** | 14.10  (1, 332) | <0.001 | **0.001** |
|  | Site | 3.09  (1, 335) | 0.080 | 0.139 | 0.31  (1, 319) | 0.578 | 0.689 | 16.58  (1, 332) | <0.001 | **<0.001** | 54.99  (1, 332) | <0.001 | **<0.001** |
|  | Education | 0.19  (1, 335) | 0.661 | 0.661 | 0.25  (1, 319) | 0.616 | 0.689 | 5.34  (1, 332) | 0.021 | 0.038 | 4.13  (1, 332) | 0.043 | 0.080 |
|  | Diabetes | 5.64  (1, 335) | 0.018 | **0.042** | 8.46  (1, 319) | 0.004 | **0.014** | 1.27  (1, 332) | 0.260 | 0.331 | 3.83  (1, 332) | 0.051 | 0.080 |
|  | Hearing loss | 0.80  (1, 335) | 0.370 | 0.471 | 1.14  (1, 319) | 0.287 | 0.670 | 0.29  (1, 332) | 0.589 | 0.589 | 0.65  (1, 332) | 0.419 | 0.489 |
| **Intensity model** | *ilrs* | 4.03  (3, 339) | 0.008 | **0.025** | 0.94  (3, 323) | 0.421 | 0.582 | 0.61  (3, 336) | 0.611 | 0.611 | 1.19  (3, 336) | 0.315 | 0.441 |
|  | Age | 0.33  (1, 339) | 0.567 | 0.661 | 0.30  (1, 323) | 0.582 | 0.582 | 8.78  (1, 336) | 0.003 | **0.008** | 0.05  (1, 336) | 0.829 | 0.829 |
|  | Sex | 160.61  (1, 339) | <0.001 | **<0.001** | 15.45  (1, 323) | <0.001 | **<0.001** | 23.30  (1, 336) | <0.001 | **<0.001** | 16.81  (1, 336) | <0.001 | **<0.001** |
|  | Site | 2.50  (1, 339) | 0.114 | 0.200 | 0.79  (1, 323) | 0.373 | 0.582 | **19.11**  (1, 336) | <0.001 | **<0.001** | 50.63  (1, 336) | <0.001 | **<0.001** |
|  | Education | 0.07  (1, 339) | 0.797 | 0.797 | 0.36  (1, 323) | 0.547 | 0.582 | 7.11  (1, 336) | 0.008 | **0.014** | 6.03  (1, 336) | 0.015 | **0.034** |
|  | Diabetes | 6.58  (1, 339) | 0.010 | **0.025** | 9.93  (1, 323) | 0.002 | **0.006** | 0.78  (1, 336) | 0.376 | 0.526 | 3.02  (1, 336) | 0.083 | 0.146 |
|  | Hearing loss | 0.45  (1, 339) | 0.505 | 0.661 | 1.25  (1, 323) | 0.265 | 0.582 | 0.40  (1, 336) | 0.526 | 0.611 | 0.42  (1, 336) | 0.517 | 0.603 |

*Note.* ANOVA F test outcomes of final models (covariate adjusted) for cardiometabolic outcomes. ilrs= isometric log-ratios used to express 24-hour time-use behaviours as a composition; ‘Domain model’ = models whereby time-use composition was created using activity superdomains as compositional parts. Bold text and asterisks indicate p-values which remained statistically significant (p<0.05) following false discovery rate adjustment. F(n,d) = F statistic, and numerator and denominator degrees of freedom.
